# Multimodal transcriptomics identifies metallothionein as a novel pathway in primary sclerosing cholangitis

**DOI:** 10.1097/HEP.0000000000001432

**Published:** 2025-06-25

**Authors:** Brian K. Chung, Markus S. Jördens, Jonas Øgaard, Henrik Mikael Reims, Benedicte Flateland, Tom Luedde, Xiaojun Jiang, Tom H. Karlsen, Espen Melum

**Affiliations:** 1 Norwegian PSC Research Center, Division of Surgery and Specialized Medicine, Department of Transplantation Medicine, Oslo University Hospital Rikshospitalet, Oslo, Norway; 2 Research Institute of Internal Medicine, Division of Surgery and Specialized Medicine, Oslo University Hospital Rikshospitalet, Oslo, Norway; 3 Institute of Clinical Medicine, Faculty of Medicine, University of Oslo, Oslo, Norway; 4 Department of Gastroenterology, Hepatology and Infectious Diseases, Medical Faculty, Heinrich Heine University Düsseldorf, University Hospital Düsseldorf, Düsseldorf, Germany; 5 Department of Pathology, Oslo University Hospital Rikshospitalet, Oslo, Norway; 6 Section of Gastroenterology, Division of Surgery and Specialized Medicine, Department of Transplantation Medicine, Oslo University Hospital Rikshospitalet, Oslo, Norway; 7 Hybrid Technology Hub, Institute of Basic Medical Sciences, Faculty of Medicine, University of Oslo, Oslo, Norway

**Keywords:** liver, primary sclerosing cholangitis, spatial transcriptomics, single-nuclei RNA sequencing, metallothionein

## Abstract

**Background and Aims:**

Primary sclerosing cholangitis (PSC) is a chronic inflammatory bile duct disorder of unknown etiology characterized by uneven peribiliary infiltration and liver fibrosis. To localize potential disease pathways to specific microanatomical liver regions, we combined spatial and single-nuclei transcriptomics (snRNA-seq) to analyze biopsies from a spectrum of PSC and disease control explants.

**Approach and Results:**

Liver specimens from 23 PSC (transplant indications: 4 recurrent cholangitis, 7 dysplasia, 12 cirrhosis) and 7 disease controls with cirrhosis (4 metabolic dysfunction–associated steatohepatitis, MASH; 3 alcohol-associated liver disease, ALD) were analyzed by spatial transcriptomics (76,664 spots) and 16 of the same explants were also assessed by snRNA-seq (12 PSC, 2 MASH, 2 ALD; 91,891 nuclei). PSC livers expressed a robust signature of metallothionein (*MT1E, MT1G*, *MT1H*) and acute inflammation markers (*SAA1*, *SAA2*) at the parenchyma–fibrosis interface compared to disease controls. SnRNA-seq showed that the strongest metallothionein signal originated from a subtype of *APOE*+ hepatocytes that, by spatial transcriptomics and immunohistochemistry, were consistently localized to the edge of fibrotic lesions in PSC explants. Biliary inflammation induced by 0.1% 3,5-diethoxycarbonyl-1,4-dihydrocollidine (DDC) feeding in mice resulted in significant *Mt1* liver expression and liver *MT1G* expression correlated with AST, ALT, ALP, and bilirubin levels at the time of liver transplantation.

**Conclusions:**

Combinatorial spatial and high-resolution single-nuclei transcriptomics on the largest number of PSC liver explants to date revealed metallothionein as a novel PSC pathway that could be interrogated in future studies aiming to develop effective therapeutic interventions for PSC.

## Graphical abstract

**Figure:**
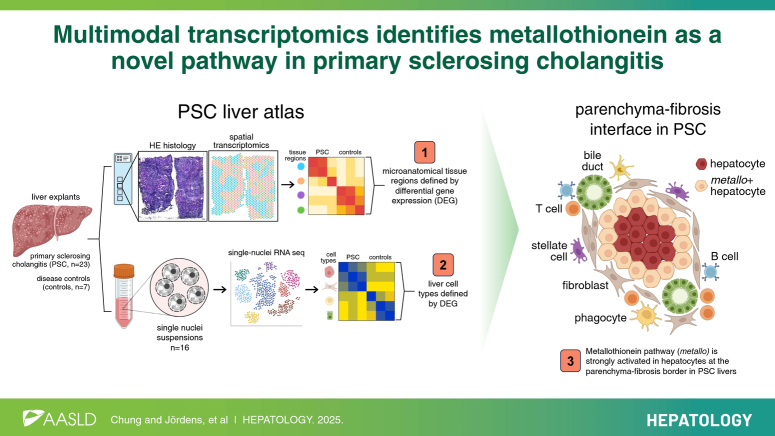

## INTRODUCTION

Primary sclerosing cholangitis (PSC) is an inflammatory cholestatic disorder characterized by fibrosis and multifocal strictures of the bile ducts.^1–3^ The disease course of PSC is progressive, with a median transplant-free survival of 13–21 years, and often leads to the development of cirrhosis or hepatobiliary cancers. Liver transplantation is the only effective treatment for PSC and is typically considered for hepatic decompensation, but also for cases with severe recurrent cholangitis, intractable cholestatic pruritus, or biliary dysplasia.^4^ The etiology of PSC is unknown, but large-scale genetic studies have shown that PSC shares a limited genetic similarity to IBD (∼50%) and unique hereditary determinants compared to ulcerative colitis and Crohn disease, primarily highlighting immune-related risk loci.^5^ Studies in patients and animal models of PSC have uncovered multiple immune cell alterations in PSC, including elevated numbers of biliary neutrophils,^6^ expansion of type 2 dendritic cells,^7^ skewing of macrophage states^8–10^ and differential abundances of T and B lymphocytes.^11–13^ Together these findings strongly support immune dysregulation as a key disease factor in PSC.

Despite these extensive efforts, a poor understanding of the underlying PSC pathophysiology remains and is in part due to the patchy and heterogenous nature of liver pathology in PSC. Characteristic obliterative periductal “onionskin” fibrosis may be present but is not pathognomonic, and portal inflammation and ductular reaction are frequent but not uniform. To dissect the patchy disease process, recent studies utilizing spatial and high-resolution single-cell RNA sequencing have highlighted multiple cell types and molecular interactions that may represent novel treatment targets. However, these studies were conducted on sorted cells,^12^ small number of patients^14,15^ or across competing platforms exclusively between PSC explants with cirrhosis and non-disease controls, limiting the discrimination of general features of end-stage liver disease from those specific to PSC.^16^

To capture a broader representation of PSC and dissect potential pathways unrelated to general end-stage liver disease signatures, we compared 23 PSC livers spanning different transplant indications (recurrent cholangitis, dysplasia, cirrhosis) and 7 non-immune disease controls (metabolic dysfunction–associated steatohepatitis, MASH; alcohol-associated liver disease, ALD) with cirrhosis by spatial transcriptomics and complemented our spatial assessment with single-nuclei RNA sequencing from 16 of the same liver explants. This multimodal approach revealed differential gene content, pathways, and cell types localized to non-fibrotic and fibrotic PSC liver regions, including a robust and novel PSC signature corresponding to a newly described subset of cholangio-hepatocytes bordering fibrotic PSC lesions that was recapitulated by inducing biliary inflammation in a cholestatic mouse model of PSC.

## METHODS

### Human samples

Fresh-frozen liver biopsies were collected from 23 PSC patients (transplant indications: 4 recurrent cholangitis, 7 dysplasia, 12 cirrhosis) and 7 disease controls (4 MASH, 3 ALD) with cirrhosis as their transplant indication were selected from the Norwegian PSC Research Center biobank. Samples were selected for inclusion after a detailed assessment of the clinical indication for transplant and liver pathology reports. Patients transplanted for recurrent PSC and overlap syndrome (PSC with autoimmune hepatitis) were excluded. Written consent was obtained from all study participants before sampling, and study ethics were approved by the regional Committees for Medical and Health Research Ethics of Southeast Norway (reference numbers: 15368 and 599798) in accordance with the Declarations of Helsinki and Istanbul.

### Tissue processing and nuclei isolation

Liver biopsies were collected within 20 minutes of liver explantation, cryopreserved, and equally divided for spatial transcriptomics and single-nuclei RNA sequencing (snRNA-seq). Tissue for spatial transcriptomics was embedded in pre-cooled Tissue-Tek optimal cutting temperature mounting medium (Sakura Finetek, Alphen aan den Rijn, Netherlands) and rapidly refrozen on dry ice for storage at −80°C. Tissue for snRNA-seq was prepared using Chromium nuclei isolation kits with RNase inhibitor (10x Genomics) according to the manufacturer’s recommendations (Rev A, 10x Genomics; see Supplemental Materials and Methods, http://links.lww.com/HEP/J842).

### Spatial transcriptomics and single-nuclei RNA sequencing

Frozen OCT-embedded explant tissue from central and peripheral liver locations was sectioned and placed on Visium RNA capture slides for spatial transcriptomics according to the manufacturer’s recommendations (10x Genomics, see Supplemental Materials and Methods, http://links.lww.com/HEP/J842). SnRNA-seq was performed using Chromium Next GEM single-cell v3.1 3’ kits (10x Genomics) following the manufacturer’s protocol (Rev E, 10x Genomics). Sequencing and pre-processing of spatial and snRNA-seq transcriptomics were completed following the manufacturer’s recommendations. Sequencing data from spatial transcriptomics and snRNA-seq can be found and explored at https://riim-data.no/NoPSC_Liver_Atlas/SPATIAL/ and https://riim-data.no/NoPSC_Liver_Atlas/SINGLE_NUC/.

### Normalization, dimensionality reduction, integration, and clustering analysis

Spatial and snRNA-seq transcripts were analyzed in RStudio using Seurat v.5.0.5.^17^ Briefly, pre-processed spatial and snRNA-seq data were normalized, and variable features were defined using SCTransform v.0.4.1.^18^ Principal component analysis of variable features was computed using the Seurat RunPCA function and integrated to correct for batch and technical artifacts using Harmony v.1.2.0.^19^ Dimensionality reduction was performed by neighbor graph-based clustering using the Seurat RunUMAP function set to 20 principal components. The number of clusters was adjusted using the resolution parameter of the Seurat FindClusters function, set between 0.05 and 4. Stable clusters were defined by at least 10 differentially expressed genes (DEGs) of significance using the differential expression output in Loupe browser v.7.0.1 (10x Genomics) detected at greater than one average count per spot (spatial) or per nuclei (snRNA-seq). DEGs of statistical significance (*p*-adjusted <0.05) were calculated in Loupe browser v.7.0.1 (10x Genomics) using the exact negative binomial test and Benjamini–Hochberg corrected for multiple testing.

### Pseudobulk, pathway enrichment analysis, and CIBERSORTx gene deconvolution

All stated DEGs between PSC and disease controls detected by spatial transcriptomics and snRNA-seq were identified using the Pseudobulk function in Loupe browser v.7.0.1 (10x Genomics) and reached statistical significance (*p*<0.01). DEGs identified by Pseudobulk were forwarded for KEGG (Kyoto Encyclopedia of Genes and Genomes) pathway enrichment (see Supplemental Materials and Methods, http://links.lww.com/HEP/J842). Spatial transcriptomes from PSC and disease controls were deconvoluted by CIBERSORTx^20^ using disease-specific snRNA-seq references generated from a subset of the same liver biopsies (12 PSC and 4 disease controls; see Supplemental Materials and Methods, http://links.lww.com/HEP/J842).

### DDC cholangitis mouse experiments and bulk RNA liver sequencing

Three-month-old C57BL/6J male mice housed in specific-pathogen-free cages were fed a 0.1% 3,5-diethoxycarbonyl-1,4-dihydrocollidine (DDC) diet (Envigo) or control chow and sacrificed at 2 and 4 weeks for liver sampling. All animal experiment protocols were approved by Mattilsynet, Norway (FOTS IDs 6519, 7033, 7821, 10426, and 14125) and conducted as recommended by the Federation of European Laboratory Animal Science Associations (FELASA). RNA from snap-frozen liver tissues was isolated and sequenced on the NovaSeq X Plus system (Illumina) in paired-end 150 bp (PE150) mode (Novogene). Raw reads were filtered and mapped to the GRCm39/mm39 *Mus musculus* reference genome, and differential gene expression of metallothionein 1 (*Mt1*) in DDC and control mouse livers was analyzed using the DESeq2 R package v1.20.0.^21^

### Immunohistochemistry and HE staining

Immunohistochemistry (IHC) staining of formalin-fixed paraffin-embedded (FFPE) human liver tissue was performed using standard protocols to detect expression of MT1G, APOE, and KRT19 (see Supplemental Materials and Methods, http://links.lww.com/HEP/J842). HE staining of central and peripheral liver tissues from PSC (n=4) and disease controls (n=2, ALD and MASH), analyzed by spatial transcriptomic, was also evaluated by an expert liver pathologist (Henrik Mikael Reims) to ascertain the concordance between tissue classification by spatial transcriptomics and morphology and pathological changes discernable by HE histology.

### Statistical analyses

All reported values indicate mean ± SD, and all standard statistical analyses were performed using Prism v10.2.2 (GraphPad) unless otherwise stated. *P*-values for comparative analysis of microanatomical fractions (pare, fibro1, fibro2) were calculated using two-way ANOVA. *P*-values for comparative analysis of spatial clusters and cell type fractions between PSC and disease controls were calculated using a two-tailed unpaired Student *t* test and Mann–Whitney *U* test. *P*-adjusted values for Pseudobulk DEG analysis were calculated by the exact negative binomial in Loupe browser (10x Genomics). *P*-values for pathway enrichment were calculated using the Fisher exact test. *R*-values and *p*-values from the analysis of *MT1G*+ parenchyma regions and clinical features were calculated using a two-tailed Spearman rank correlation test.

## RESULTS

### Spatial transcriptomics classifies distinct microanatomical regions in central and peripheral liver biopsies

**FIGURE 1:**
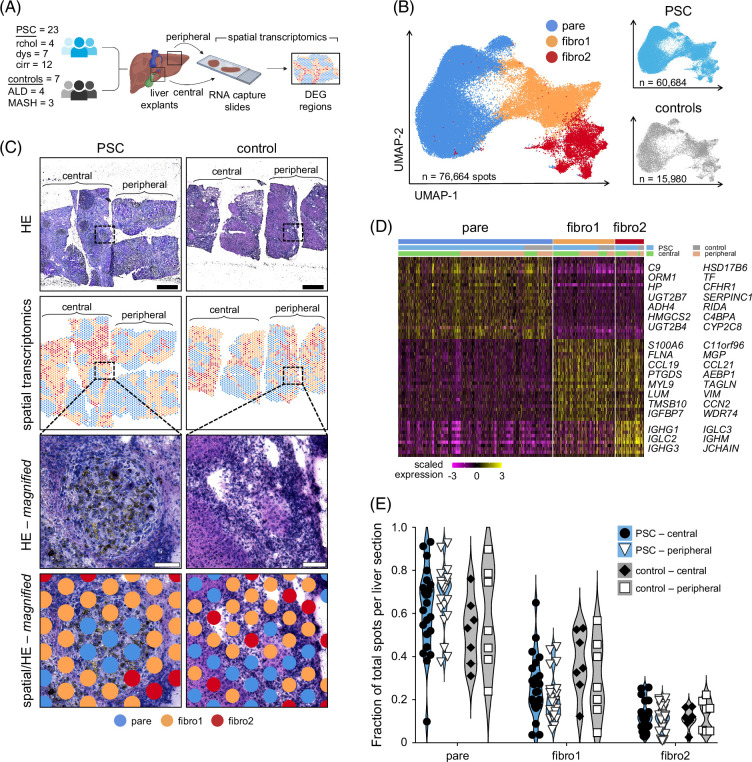
Classification of microanatomical liver regions by spatial transcriptomics. (A) Overview of study cohort and spatial transcriptomics workflow. (B) UMAPs indicate microanatomical classification of RNA capture spots by spatial transcriptomics from all PSC and disease control livers and are split by disease indication. (C) Representative HE staining and spatial transcriptomics of liver sections from PSC and disease control explants. Dotted boxes indicate magnified regions. Black scale bars=1 mm, white scale bars in magnified images=100 µm. (D) Heatmap of top DEGs per microanatomical cluster. Rows indicate genes, columns represent RNA capture spots grouped by microanatomical classification (pare, fibro1, fibro2), disease indication (PSC, control), and sampling location (central, peripheral). (E) Fraction of total RNA capture spots classified as pare, fibro1, and fibro2 by spatial transcriptomics per central or peripheral liver section. Statistical significance was evaluated by two-way ANOVA. Abbreviations: cirr, cirrhosis; controls, disease controls; DEG, differentially expressed gene; dys, dysplasia; fibro1, fibrosis1; fibro2, fibrosis2; HE, hematoxylin and eosin; MASH, metabolic dysfunction–associated steatohepatitis; pare, parenchyma; rchol, recurrent cholangitis; UMAP, uniform manifold approximation and projection.

To molecularly characterize disease processes and generate a comprehensive dataset enabling integrated assessments, we used spatial transcriptomics to analyze paired central and peripheral liver biopsies from 23 PSC patients (transplant indications: 4 recurrent cholangitis, 7 dysplasia, 12 cirrhosis) and 7 disease controls (4 MASH, 3 ALD) with cirrhosis as their transplant indication (Figure 1A and Supplemental Table S1, http://links.lww.com/HEP/J842). Tissue samples were collected by punch biopsy within 20 minutes of liver explantation and selected for study inclusion based on detailed assessment of clinical indication for transplant and liver pathology reports. Patients with recurrent PSC or AIH/PSC overlap were excluded. Median age of the PSC cohort was 42 years (range: 29–67 years) and 62 years (range: 55–70 years) for disease controls. PSC patients and disease controls both had a male predominance (PSC: 17/23, controls: 5/7), and 16/23 of the PSC patients had concomitant IBD compared to 0/7 disease controls.

To define comparable microanatomical liver regions by spatial transcriptomics, we normalized, integrated, and partitioned spatial transcripts from every sample (n=30, 76,664 total spots) into 3 distinct clusters (parenchyma, fibrosis1, and fibrosis2) based on differential gene expression (Figure 1B). Discrimination of the parenchyma and fibrosis by spatial transcriptomics closely aligned with histological features observed by hematoxylin and eosin (HE) staining as we have previously reported (Figure 1C).^15^ Parenchyma (pare) gene content was dominated by known hepatocyte markers (*TF*, *HP*, *ALB*) whereas fibrosis1 (fibro1) was enriched for fibroblast and stellate cell markers (*C11orf96*, *IGFBP*, *LUM*, *MYL9, PTGDS*) as well as endothelial cells (*MGP*, *CCL21*). Gene profiles of fibrosis2 (fibro2) closely resembled fibro1 but clearly segregated due to approximately a 4-fold higher expression of B cell Ig genes in fibro2 versus fibro1 (Figure 1D). Ig expression was also detected in pare but was 8-fold lower than fibro2, indicating that B cells localize preferentially to regions of liver fibrosis.

To determine if potential pathogenic pathways differed between microanatomical regions within the same liver, we compared the gene content of pare, fibro1, and fibro2 in central and peripheral biopsies from the same PSC or disease control explants. We observed no significant difference in spatial content within the same microanatomical region of central and peripheral samples, implying that major disease processes within the same liver are largely consistent (Supplemental Figure S1, http://links.lww.com/HEP/J842). Proportions of each microanatomical region were similar between central and peripheral biopsies, irrespective of disease (Figure 1E) or transplant indication for PSC patients (Supplemental Figure S2, http://links.lww.com/HEP/J842). Evaluation of the HE stained slides from 4 PSC and 2 disease control livers (ALD and MASH) confirmed that microanatomical regions classified as parenchyma (pare) or fibrosis (fibro1 + fibro2) by spatial transcriptomics (1684 total spots assessed) were 96.3% concordant with morphological features and pathological changes discernable by HE histology (Supplemental Figure S3, http://links.lww.com/HEP/J842). Together, these findings demonstrate that gene pathways and the ratio of parenchyma to fibrosis across each liver explant are comparable in PSC and disease control explants as evaluated by spatial transcriptomics.

### Non-cirrhotic PSC liver is characterized by reduced cholangiocytes and activation of the metallothionein pathway

**FIGURE 2:**
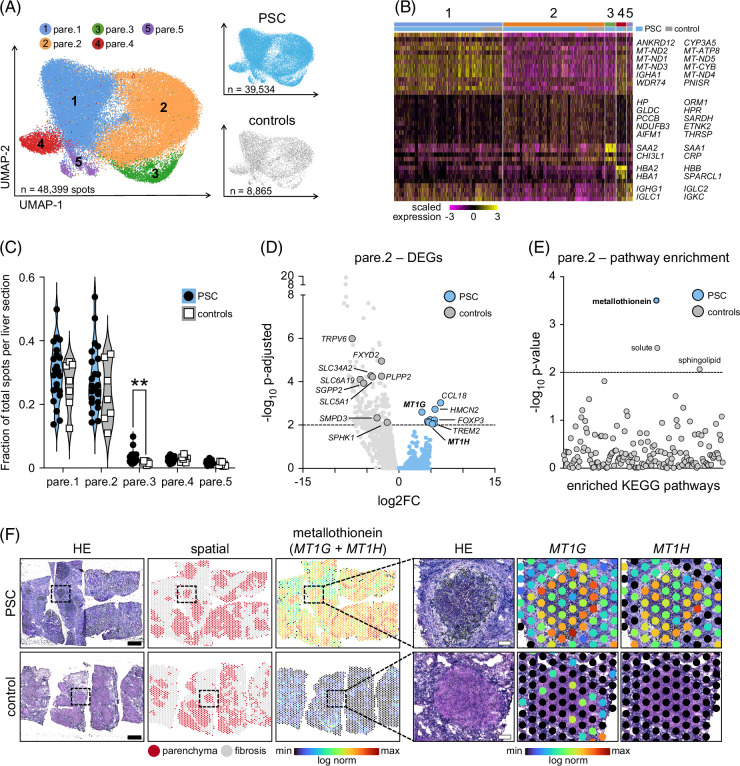
Metallothionein expression is significantly elevated in the liver parenchyma of patients with PSC. (A) UMAPs show subclustering of liver parenchyma from all PSC and disease control explants defined by spatial transcriptomics. RNA capture spots classified as pare.1–pare.5 and annotated by disease indication. (B) Heatmap of top DEGs per cluster. Rows indicate genes, columns represent RNA capture spots grouped by pare.1–pare.5 and disease indication (PSC, control). (C) Fraction of total RNA capture spots classified as pare.1–pare.5 per liver section. (D) DEG analysis of pare.2 from PSC and disease controls. (E) KEGG pathway enrichment analysis of significant DEGs in pare.2 of PSC and disease controls. (F) Spatial transcriptomics of metallothionein (*MT1G* + *MT1H*) in PSC and disease control liver sections. Dotted boxes indicate magnified regions. Black scale bars=1 mm, white scale bars in magnified images=100 µm. Statistical significance was evaluated for (C) using the Mann–Whitney *U* test and unpaired Student *t* test, for (D) using the exact negative binomial test, and for (E) using the Fisher exact test. Dotted lines in (D) and (E) indicate *p*-adjusted=0.01 and *p*=0.01. Abbreviations: Controls, disease controls; DEGs, differentially expressed genes; HE, hematoxylin and eosin; KEGG, Kyoto Encyclopedia of Genes and Genomes; log norm, log normalized; pare.1–pare.5, parenchyma subclusters 1–5; PSC, primary sclerosing cholangitis; UMAP, uniform manifold approximation and projection.

After dissecting each liver section into microanatomical regions, we assessed the parenchyma (pare) classified by spatial transcriptomics to identify potential PSC pathways within non-fibrotic liver regions. As we detected similar gene content in central and peripheral biopsies, pare spots from every PSC and disease control were reclustered together (48,399 spots), which revealed 5 parenchyma subclusters (pare.1–pare.5; Figure 2A). Pare.1 featured high mitochondrial gene expression indicative of apoptotic or dying cells, whereas pare.2 was characterized by markers of hepatocyte function (*HP*, *ORM1*), cellular metabolism (*GLDC*, *PCCB*, *SARDH*), and fatty acid degradation (*GCDH*, *ACAT2*; Figure 2B and Supplemental Table S2, http://links.lww.com/HEP/J842). DEGs in pare.3 related to acute-phase inflammation (*SAA1*, *SAA2*, *CHI3L1*, *CRP*), DEGs in pare.4 associated with erythrocytes and hemoglobin binding (*HBA1*, *HBB*, *HBA1*) and DEGs in pare.5 represented B cells (*IGHG1*, *IGKC, IGLC2*, *IGLC1*). Relative fractions of each pare subcluster per liver were comparable between PSC and disease controls, except for pare.3, where higher proportions were seen in PSC explants, suggesting that PSC may be associated with greater acute-phase inflammation in the parenchyma compared to non-PSC disease controls (Figure 2C).

Next, we assessed each parenchyma subcluster for enriched DEGs in PSC versus controls by normalizing gene values to the number of parenchymal spots per biopsy using Pseudobulk. Overrepresented DEGs for PSC were only detected in pare.2 (n=10; Figure 2D) and featured markers of the metallothionein mineral absorption pathway (*MT1G* and *MT1H*; Figure 2E) and of scar-associated liver monocytic phagocytes (MPs: *CCL18*, *OTOA*, *TREM2*).^22^ By contrast, 330 DEGs were overrepresented in pare.2 for controls and related to cholangiocytes (*EPCAM*, *KRT19*, *SPINT1*, *SFRP5*, *SYT8*) and pathways regulating solute mineral absorption (*FXYD2*, *SLC34A2*, *SLC5A1*, *SLC6A19*, *TRPV6*) and sphingolipid metabolism (*SGPP2*, *SMPD3*, *SPHK1*, *PLPP2*). DEGs typical of cholangiocytes (*FXYD2*, *SFRP5*, *CLDN10*, *PROM1*; Supplemental Table S3, http://links.lww.com/HEP/J842) were also overexpressed in pare.1 (n=5) and pare.3 (n=12) of disease controls. DEG analysis of each parenchymal subcluster between patients with early-stage PSC (transplanted because of recurrent cholangitis or dysplasia on ERCP brush cytology, n=11) and patients with typical end-stage PSC transplanted for cirrhosis (n=12) identified 109 DEGs in pare.2 and 47 DEGs in pare.3 (Supplemental Figure S4, http://links.lww.com/HEP/J842); however, gene enrichment analysis did not detect any overlapping parenchymal pathways unique to early or end-stage PSC (Supplemental Figure S5, http://links.lww.com/HEP/J842).

Spatial mapping of metallothionein markers in PSC pare.2 revealed the greatest enrichment of *MT1G* and *MT1H* at the parenchyma–fibrosis interface of PSC livers (Figure 2F) whereas solute (*FXYD2, SLC34A2*, *SLC5A1*, *SLC6A19*, *TRPV6*) and sphingolipid markers (*DEGS2*, *PLPP2*, *SGPP2*, *SMPD3*, *SPHK1*) overrepresented in disease controls were found throughout the parenchyma and fibrosis (Supplemental Figure S6, http://links.lww.com/HEP/J842). These findings suggest that metallothionein expression, scar-associated MPs, and ductopenia may be associated with non-fibrotic PSC pathogenesis.

### High metallothionein content localizes to the parenchyma–fibrosis interface in PSC livers

**FIGURE 3:**
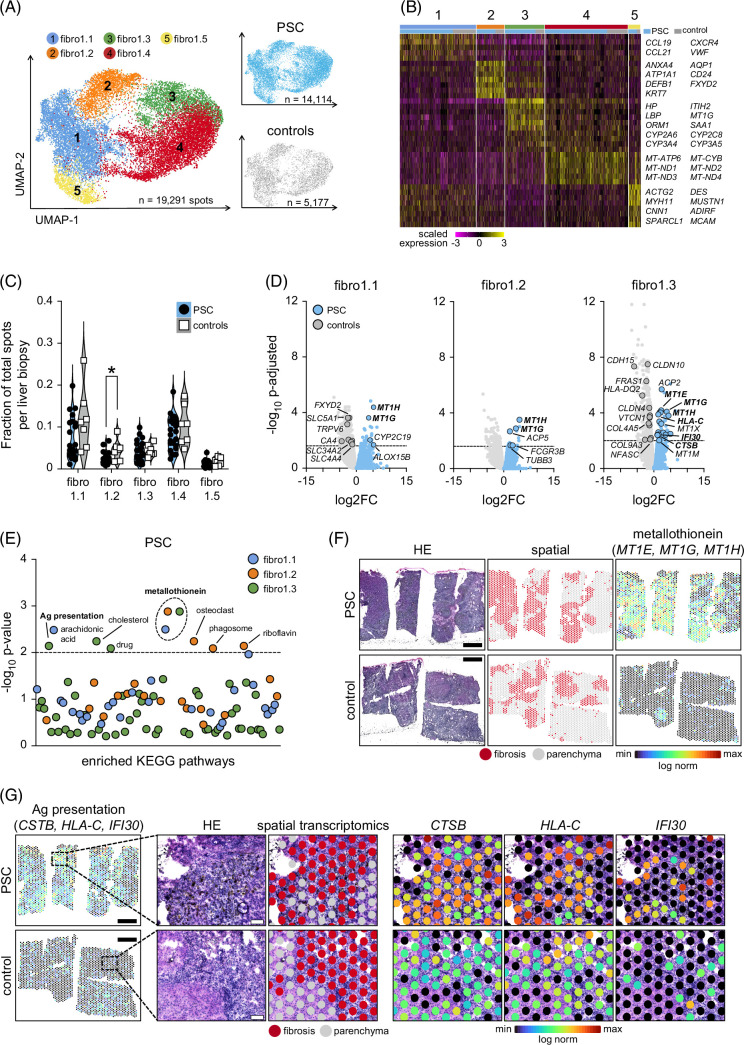
Fibrotic PSC liver regions contain fewer cholangiocyte areas and increased markers of antigen presentation. (A) UMAPs show subclustering of fibro1 from all PSC and disease control explants defined by spatial transcriptomics. RNA capture spots classified as fibro1.1–fibro1.5 and annotated by disease indication. (B) Heatmap of top DEGs per cluster. Rows indicate genes, columns represent RNA capture spots grouped by fibro1.1–fibro1.5 and disease indication. (C) Fraction of total RNA capture spots classified as fibro1.1–fibro1.5 per liver section. (D) DEG analysis of fibro1.1, fibro1.2, and fibro1.3 from PSC and disease controls. (E) KEGG pathway enrichment analysis of significant DEGs in fibro1.1, fibro1.2, and fibro1.3 of PSC and disease controls. (F) Spatial transcriptomics of metallothionein (*MT1G* and *MT1H*) in PSC and disease control liver sections. Black scale bars=1 mm. (G) Spatial transcriptomics of antigen presentation markers (*CTSB*, *HLA-C*, *IFI30*) in PSC and disease control liver sections. Dotted boxes indicate magnified regions. Black scale bars=1 mm, white scale bars in magnified images=100 µm. Statistical significance was evaluated for (C) using the Mann–Whitney *U* test and unpaired Student *t* test, for (D) using the exact negative binomial test, and for (E) using the Fisher exact test. Dotted lines in (D) and (E) indicate *p*-adjusted=0.01 and *p*=0.01. *p<0.05. Abbreviations: controls, disease controls; fibro1.1–fibro1.5, fibrosis1.1–fibrosis1.5; HE, hematoxylin and eosin; PSC, primary sclerosing cholangitis; UMAP, uniform manifold approximation and projection.

We next sought to investigate gene pathways localized to fibrotic regions in PSC and first interrogated fibro1, which constituted the largest fibrosis cluster (19,291 of 28,265 total fibrosis spots) and differed from fibro2 primarily due to low Ig expression (Figures 1B, D). To interrogate fibro1, we reclustered all fibro1 spots from central and peripheral PSC and control biopsies and detected 5 subsets with distinct DEG profiles (fibro1.1–fibro1.5; Figure 3A and Supplemental Table S4, http://links.lww.com/HEP/J842). Fibro1.1 featured DEGs relating to periportal lymphatic endothelial cells (*CCL19*, *CCL21*, *VWF*), B cells (*IGHA1*, *IGHM*, *JCHAIN*), and leukocyte migration (*CXCR4, CXCL12*) whereas DEGs in fibro1.2 related to cholangiocytes (*ANXA4*, *AQP1*, *DEF1, FXYD2*, *DEFB1*, *KRT7*), bicarbonate secretion and absorption (*ATP1A1*, *AQP1*, *FXYD2*; Figure 3B). Several DEGs overexpressed in fibro1.3 overlapped with those in pare.2, including genes relating to metallothionein (*MT1E*, *MT1G*, *MT1X*, *MT2A*) and hepatocyte metabolism (*HP, ORM1*) as well as drug detoxification (*CYP2A6*, *CYP2C8*, *CYP34*, *CYP3A5*) and acute-phase inflammation (*SAA1, SAA2, SAA4*). Spatial mapping of fibro1.3 and pare.2 showed these clusters reside in close tissue proximity (Supplemental Figure S7, http://links.lww.com/HEP/J842). DEGs in fibro1.4 were primarily mitochondrial in origin (8 of the top 10), with 7 of the 8 corresponding to the top 10 DEGs in pare.1.1 suggesting that both clusters represented areas of apoptotic and dying cells. Fibro1.5 was the smallest fibro1 cluster (969 spots) with DEGs associated with smooth muscle cells (*ACTG2*, *CNN1*, *DES*, *MUSTN1*) and pericentral endothelial markers absent from fibro1.1 (*ADIRF*, *MCAM*, *PECAM*).^23^ Abundance analysis showed that fibro1.2 spots featuring mostly cholangiocyte markers were significantly reduced in PSC (Figure 3C), while a trend toward fewer fibro1.1 spots was also seen in PSC explants (*p*=0.068). Mean proportions of cholangiocytes (KRT19+ cells) quantified by IHC staining of liver tissue from 27 of 30 individuals assessed by spatial transcriptomics (PSC: 20, disease controls: 7) were reduced in PSC livers versus disease controls (PSC: 0.035, disease controls: 0.135; *p*<0.0001; Supplemental Figure S8, http://links.lww.com/HEP/J842) which supports that cholangiocyte-rich regions reduced in PSC parenchyma are also decreased in PSC fibrosis as measured by spatial transcriptomics. No differences in spatial spots classified as fibro1.3 (hepatocytes), fibro1.4 (apoptotic/dying cells), and fibro1.5 (pericentral regions) were seen between PSC and disease controls.

After classifying comparable regions in liver fibrosis, we analyzed each fibro1 subcluster by Pseudobulk (Figure 3D) to identify potential upregulated disease pathways in PSC fibrosis. We detected a limited number of DEGs overexpressed in PSC for fibro1.1 (n=28), fibro1.2 (n=18), and fibro1.3 (n=59). Greater numbers of DEGs were upregulated in controls compared to PSC, including 243 in fibro1.1, 96 in fibro1.2, 399 in fibro1.3, 20 in fibro1.4, and 3 in fibro1.5 (Supplemental Table S5, http://links.lww.com/HEP/J842). Although fewer in number, overrepresented DEGs in PSC corresponded to a greater number of significantly enriched pathways (n=8) compared to controls (n=4) and again implicated metallothionein as a key PSC disease pathway in line with our findings in the parenchyma (Figure 3E). Additional pathways enriched in PSC fibro.1 and not seen in PSC parenchyma related to immune activation, including phagocytosis (fibro1.2: *FCGR3B*, *TUBB3*), antigen presentation (fibro1.3: *CTSB*, *HLA-C*, *IFI30*), metabolism of inflammatory mediators (fibro1.1: arachidonic acid: *ALOX15B*, *CYP2C19*; fibro1.2: riboflavin: *ACP5*) and scar remodeling (fibro1.2: osteoclastogenesis: *FCGR3B*, *ACP5*). Pathways most strongly enriched in controls again related to those identified in the spatial analysis of the parenchyma, including solute mineral absorption (*SLC34A2*, *SLC6A19*, *MT1A*, *TRPV6*, *FXYD2*, *SLC5A1*) and bicarbonate reclamation (*FXYD2*, *CA4*, *SLC4A4*) whereas pathways enriched in controls and found only in fibro1 related to cellular adhesion (fibro1.3: *CLDN11*, *CLDN10*, *CLDN4*, *NFASC*, *ITGB8*, *VTCN1*, *CDH15*, *HLA-DQA2*) and extracellular matrix–receptor interactions (fibro1.3: *TNXB*, *FRAS1*, *VWF*, *ITGB8*, *COL4A5*, *COL9A3*). DEG analysis of each fibro1 subcluster between patients with early-stage and end-stage PSC revealed 435 DEGs in fibro1.3 (Supplemental Figure S4, http://links.lww.com/HEP/J842). Upregulated DEGs in early-stage PSC were related to 19 enriched pathways ranging from bile secretion to amino acid degradation, whereas DEGs in end-stage PSC were strongly related to pathways involved in the regulation of fibrosis (Supplemental Figure S5, http://links.lww.com/HEP/J842).

As expected, spatial mapping of PSC-enriched pathways showed the strongest expression of metallothionein (*MT1E*, *MT1G*, *MT1H*) at the parenchyma–fibrosis interface, whereas markers for antigen presentation (*CSTB*, *HLA*-C, *IFI30*) were distributed throughout fibrotic septa (Figures 3F, G). Expression of the strongest enriched pathways in controls (mineral absorption: *FXYD2*, *SLC34A1*, *SLC5A1*, *SLC6A19*, *TRPV6*) was also regionalized to the parenchyma–fibrosis interface (Supplemental Figure S9, http://links.lww.com/HEP/J842). Collectively, these results highlight metallothionein and immune activation as potential pathogenic networks in PSC fibrosis and the parenchyma–fibrosis interface as a heightened neighborhood of active disease in both PSC and disease controls.

### B cells and plasma cells infiltrate multiple liver compartments in PSC and disease controls

Lastly, we addressed the B cell infiltrated fibrotic areas and reclustered all fibro2 spots (n=8974) initially classified as distinct based on high Ig content. Reclustering revealed 10 fibro2 subsets largely distinguished by the exclusive expression of individual Igs (eg, *IGHM*, *IGHA1*, *IGHG1*, etc) however 4 subclusters featured regional markers relating to periportal lymphatic endothelia (*CCL19*, *CCL20*) and cholangiocytes (*DEFB1*, *FXYD2*), while 2 clusters related to hepatocytes (*HP*, *ORM1*, *ORM2*) and apoptotic/dying cells (*MT* genes, Supplemental Figure S10A, http://links.lww.com/HEP/J842).

To determine if gene content differed between fibro2 periportal or hepatocyte regions, we merged and reclustered neighboring subsets into periportal (fibro2.1), hepatocytes (fibro2.2), and apoptotic/dying cells (fibro2.3) and detected over 2-fold greater Ig content in periportal neighborhoods compared to hepatocyte and apoptotic/dying cell regions (Supplemental Figure S10B, http://links.lww.com/HEP/J842). Abundance analysis showed that periportal regions (fibro2.1) constituted the largest fraction of total liver spots, but no difference was observed in relative fractions between PSC and disease controls (Supplemental Figure S11A, http://links.lww.com/HEP/J842). Differential gene analysis between each fibro2 cluster from PSC and disease controls revealed 16 total DEGs for periportal regions, 327 for the hepatocyte cluster (fibro2.2), and none for apoptotic/dying cells (fibro2.3, Supplemental Figure S11B, http://links.lww.com/HEP/J842). Multiple genes and pathways enriched in PSC fibro1 were also overrepresented in PSC fibro2 (8/14 fibro2 pathways), including several markers of the metallothionein pathway (*MT1E*, *MT1G*, *MT1H*, *MT1X*, *MT2A;* Supplemental Figure S11C, http://links.lww.com/HEP/J842). DEG analysis of each fibro2 subcluster between patients with early-stage and end-stage PSC revealed 182 DEGs in fibro2.2 (Supplemental Figure S4, http://links.lww.com/HEP/J842). Pathways relating to DEGs in early-stage PSC largely overlapped with those in fibro1.3, including bile secretion, cholesterol metabolism, peroxisome proliferator-activated receptor-γ (PPARγ) signaling, and steroid biosynthesis, whereas DEGs in end-stage PSC again related to pathways involved in the regulation of fibrosis (Supplemental Figure S5, http://links.lww.com/HEP/J842). Overall, these findings show that B cells and plasma cells preferentially reside in periportal regions of inflamed livers, and fibro2 DEGs in PSC and disease controls closely resemble those of fibro1 as analyzed by spatial transcriptomics.

### SnRNA-seq reveals alterations in cell abundance and gene content in PSC livers

**FIGURE 4:**
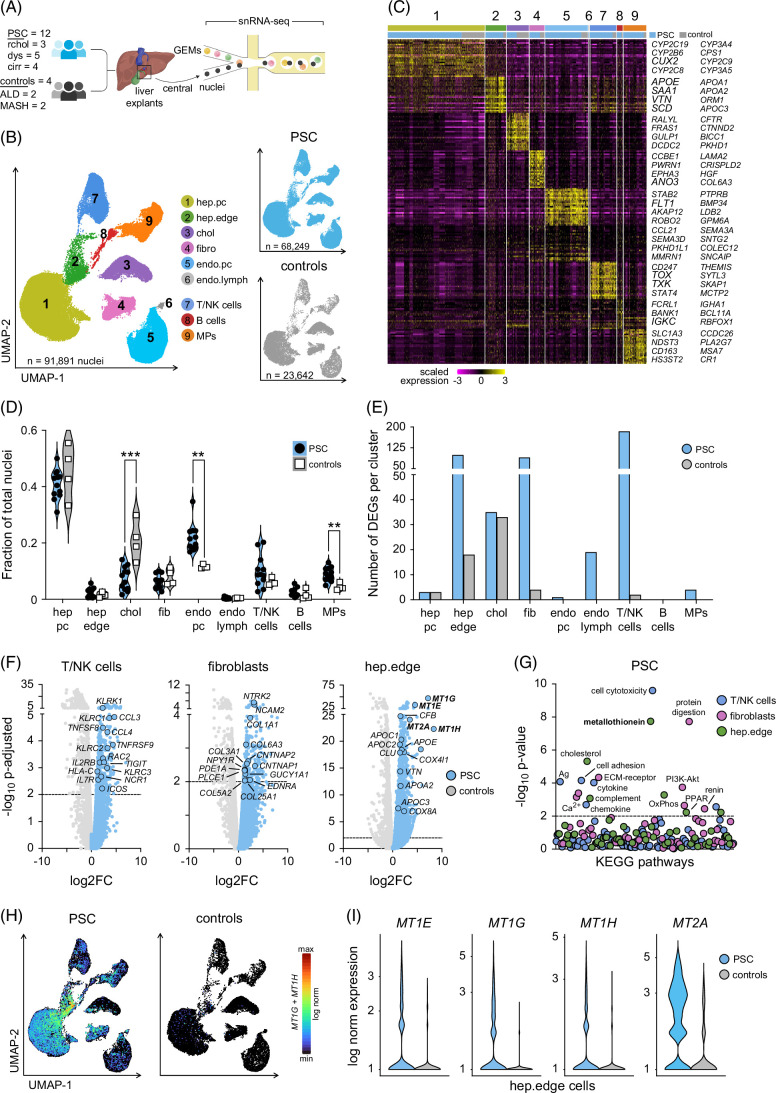
SnRNA-seq identifies altered cell abundances and gene content in PSC livers. (A) Overview of snRNA-seq study cohort and workflow. (B) UMAPs indicate snRNA-seq cell types from all PSC and disease control livers and cells split by disease indication. (C) Heatmap of top DEGs per cluster. Rows indicate genes, columns represent cell type clusters 1–9, and disease indication. (D) Fraction of each cell type as proportion of total cells per sample. (E) Total number of DEGs per cell type in PSC versus disease controls. (F) DEG analysis of T/NK cells, fibroblasts, and hep.edge cells from PSC versus disease controls. (G) KEGG pathway enrichment analysis of significant DEGs in T/NK cells, fibroblasts, and hep.edge from PSC livers. (H) UMAPs indicate *MT1G* and *MT1H* expression measured by snRNA-seq per cell type split by disease indication. (I) Relative *MT1E*, *MT1G*, *MT1H*, *MT2A* expression in PSC versus disease control hep.edge cells. Statistical significance was evaluated for (D) using an unpaired *t* test, for (F) using an exact negative binomial test, and for (G) using the Fisher exact test. Dotted lines in (F) and (G) indicate *p*-adjusted=0.01 and *p*=0.01. **p<0.01 and ***p<0.001. Abbreviations: ALD, alcohol-associated liver disease; chol, cholangiocytes; cirr, cirrhosis; controls, disease controls; DEGs, differentially expressed genes; dys, dysplasia; endo.lymph, lymphatic endothelia; endo.pc, pericentral endothelia; FC, fold-change; fib, fibroblast; GEMs, gel bead-in-emulsion; hep.edge, edge hepatocytes; hep.pc, pericentral hepatocytes; KEGG, Kyoto Encyclopedia of Genes and Genomes; log norm, log normalized; MPs, monocytic phagocytes; NK, natural killer; PSC, primary sclerosing cholangitis; rchol, recurrent cholangitis; snRNA-seq, single-nuclei RNA sequencing; UMAP, uniform manifold approximation and projection.

As spatial gene mapping has been shown to be improved when combined with complementary single-cell methodologies,^15^ we performed snRNA-seq on 91,891 nuclei isolated from 16 of the same 30 explants assessed by spatial transcriptomics (12 PSC and 4 disease controls; Figure 4A). SnRNA-seq revealed 9 major clusters that were annotated into cell types using the top 10 DEGs per cluster referenced against canonical lineage and PSC liver disease markers (Figure 4B, C and Supplemental Table S6, http://links.lww.com/HEP/J842).^16,22,24–27^ Enumeration of each cell cluster as a fraction of total nuclei per sample showed 2-fold greater proportions of pericentral endothelia and mononuclear phagocytes (MPs) and 2-fold lower fractions of cholangiocytes in PSC explants versus disease controls (Figure 4D). These findings are consistent with previous reports that endothelia and MPs contribute to liver cirrhosis^16,22,24^ and support our spatial results showing that biliary regions are damaged in PSC.

To assess if specific molecular pathways were elevated in PSC, we compared each snRNA-seq cell type from PSC and disease controls by Pseudobulk and detected the greatest enrichment of DEGs in PSC T cells and natural killer (NK) cells (T/NK, n=184; Figure 4E). T and NK cell DEGs related to cytotoxicity and antigen presentation (*KLRC1*, *KLRC2*, *KLRC3*, *KLRC4*, *HLA-C*, *HLA-F*) as well as chemokine and cytokine signaling (*CCL3*, *CCL4*, *CCLRL2*, *CXC3CR1*, *IL7R*, *IL27RA*) supporting that multiple immune alterations may act as disease factors in PSC (Figures 4F, G).^5^ Enriched DEGs were also found in PSC fibroblasts (fib, n=89) and in PSC “edge” hepatocytes (hep.edge, n=99), a newly described hepatocyte subset that co-express cholangiocyte markers and border the leading edge of fibrotic scars.^16^ Fibroblast DEGs were indicative of collagen deposition (*COL1A1*, *COL3A1*, *COL5A2*, *COL6A3*) and calcium and cAMP pathways (*EDNRA*, *F2R*, *PLCE1*, *PTGER3*), which are known to regulate key fibrosis pathways.^28^ Strikingly, DEGs in hep.edge are clearly related to cholesterol metabolism (*APOA2*, *APOC2*, *APOC2*, *APOE*) and metallothionein (*MT1E*, *MT1F*, *MT1G*, *MT1H*, *MT2A*), 2 of the strongest PSC signatures repeatedly detected in our spatial analysis. SnRNA-seq confirmed that PSC hep.edge expressed the greatest metallothionein content (*MT1E*, *MT1G*, *MT1H*, *MT2A*); however, PSC hep.pc also expressed high levels of metallothionein, and both were dramatically elevated in metallothionein compared to hepatocytes of disease controls (Figures 4H, I).

### Regions of high metallothionein content border the parenchyma–fibrosis interface in close proximity to cholangiocyte regions

**FIGURE 5:**
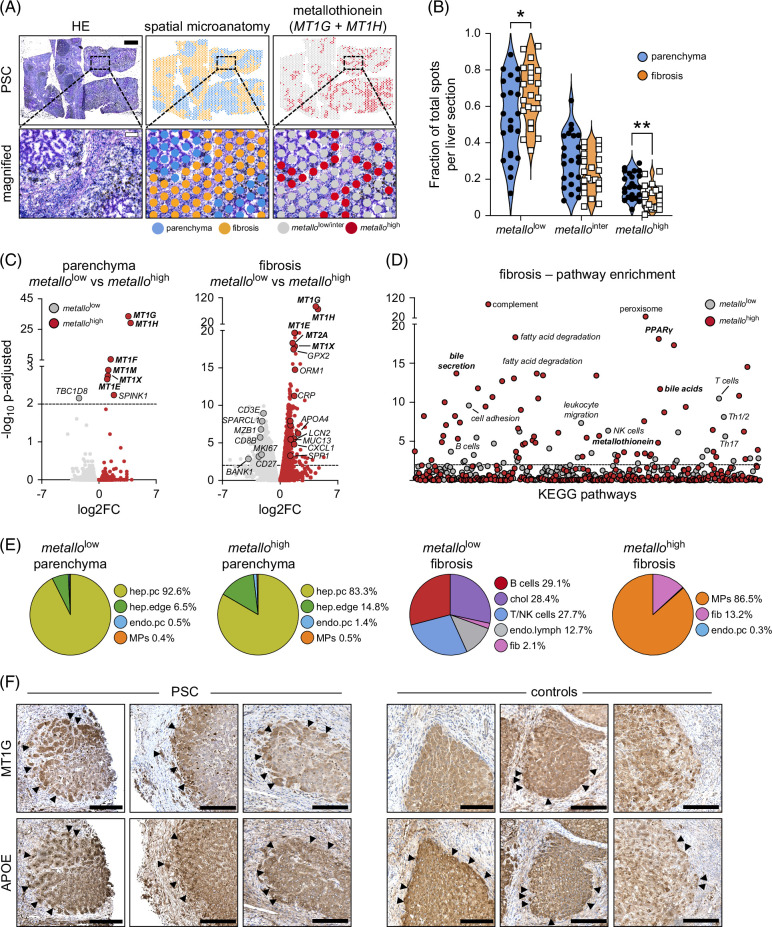
Strongest metallothionein expression localizes to the parenchyma–fibrosis interface in livers of patients with PSC. (A) Representative HE staining and *MT1G* and *MT1H* spatial transcriptomics of PSC liver section. Dotted boxes indicate magnified regions. Black scale bar=1 mm, white scale bar in magnified image=100 µm. (B) Fraction of total RNA captures spots from PSC livers classified as *metallo* ^low^, *metallo* ^inter^, and *metallo* ^high^ by spatial transcriptomics. (C) DEG analysis of *metallo* ^low^ versus *metallo* ^high^ in parenchyma and fibrosis regions defined by spatial transcriptomics. (D) KEGG pathway enrichment analysis of significant DEGs in *metallo* ^low^ versus *metallo* ^high^ fibrosis of PSC livers. (E) Estimated proportion of cell types in *metallo* ^low^ and *metallo* ^high^ spots within the parenchyma or fibrosis of PSC livers classified by spatial transcriptomics using disease-matched snRNA-seq references and CIBERSORTx deconvolution. (F) Representative IHC staining of MT1G and APOE for PSC and disease control liver sections. Arrows show positive staining, scale bars=1 mm. Statistical significance was evaluated for (B) using the Mann–Whitney *U* test, for (C) using the exact negative binomial test, and for (D) using the Fisher exact test. Dotted lines in (C) and (D) indicate *p*-adjusted=0.01 and *p*=0.01. *p<0.05 and **p<0.01. Abbreviations: Chol, cholangiocytes; endo.lymph, lymphatic endothelia; endo.pc, pericentral endothelia; fib, fibroblast; HE, hematoxylin and eosin; hep.edge, edge hepatocytes; hep.pc, pericentral hepatocytes; inter, intermediate; KEGG, Kyoto Encyclopedia of Genes and Genomes; log2FC, log2 fold-change; *metallo*, combined *MT1G* and *MT1H* expression; MPs, monocytic phagocytes; NK, natural killer; PSC, primary sclerosing cholangitis.

As spatial transcriptomics and snRNA-seq of PSC livers independently highlighted metallothionein as a potential PSC-relevant disease pathway, we integrated the spatial transcriptomics and snRNA-seq datasets to describe the cellular composition of metallothionein-rich regions. To identify areas of high metallothionein, we divided each liver biopsy into tertiles using combined *MT1G* and *MT1H* normalized spatial values and observed that regions with the highest metallothionein (*metallo* ^high^) precisely coincided with the parenchyma–fibrosis interface and areas with the lowest (*metallo* ^low^) were more frequent in fibrosis (Figures 5A, B). No differences were observed in the expression pattern and levels of *metallo* expression between male and female individuals with PSC (data not shown). Pseudobulk analysis of DEGs in *metallo* ^high^ and *metallo* ^low^ regions in PSC livers defined as parenchyma by spatial transcriptomics revealed few DEGs (8 total, Figure 5C), whereas gene content between *metallo* ^high^ and *metallo* ^low^ regions in fibrosis differed substantially, with 755 upregulated genes detected in *metallo* ^high^ and 750 genes overexpressed in *metallo* ^low^ regions. Gene enrichment analysis of *metallo* ^high^ fibrosis in PSC livers identified 57 enriched pathways, including metallothionein, bile secretion, bile acid biosynthesis, PPARγ signaling, and multiple pathways related to energy, lipid, and amino acid metabolism (Figure 5D). Gene enrichment analysis of *metallo* ^low^ regions in fibrosis of PSC livers identified 30 enriched pathways primarily associated with the differentiation and intracellular signaling of immune cells. CIBERSORTx deconvolution of *metallo* regions with our snRNA-seq PSC atlas showed that hepatocytes with high MT content (hep.edge) were enriched 2-fold in *metallo* ^high^ regions compared to *metallo* ^low^ (Figure 5E). Fibrotic *metallo* ^low^ regions conversely were dominated by lymphocytes (B cell, T cell, NK cell), cholangiocytes, and lymphatic endothelia, with the exception of MPs, which were highly enriched near active fibroblasts (*metallo* ^high^ fibrosis). To confirm our spatial results, we used APOE expression as a marker of hep.edge cells and observed by immunohistochemistry that *metallo*+ hep.edge cells were enriched at the parenchyma–fibrosis interface compared to disease controls (Figure 5F). Together, these findings suggest that hep.edge hepatocytes are enriched at the parenchyma–fibrotic interface and express higher levels of metallothionein in PSC livers compared to disease controls.

### Metallothionein expression correlates with biliary inflammation in mice and serological markers of liver disease at the time of transplant in patients

**FIGURE 6:**
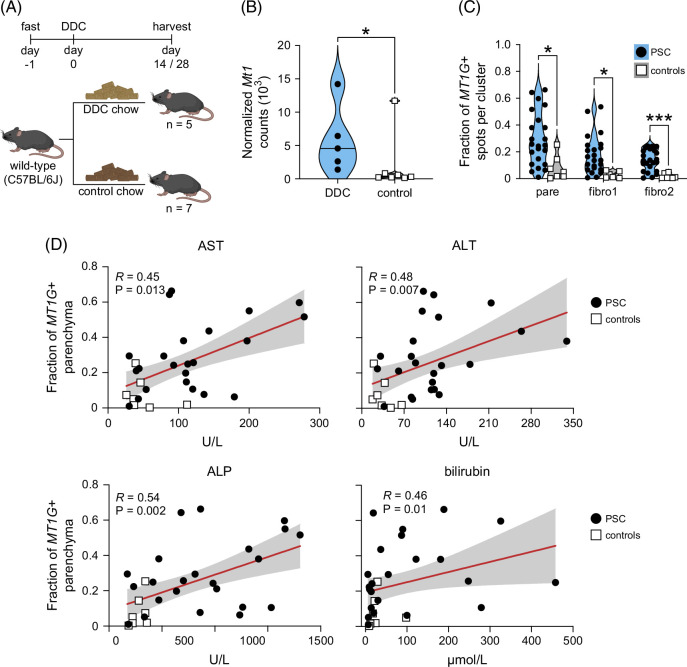
Metallothionein expression correlates with biliary inflammation in mice and serological markers at the time of liver transplantation in patients. (A) Overview of the DDC diet mouse experiment. (B) Normalized *Mt1* liver expression in mice fed 0.1% DDC diet or regular chow. (C) Fractions of *MT1G*+ RNA capture spots within pare, fibro1, or fibro2 liver regions of PSC and disease control explants classified by spatial transcriptomics. (D) Correlation analysis of *MT1G*+ parenchyma and indicated biochemical serology markers in PSC and disease controls. Statistical significance was evaluated for (B) using an unpaired Student *t* test, for (C) using the Mann–Whitney *U* test, and for (D) using Spearman rank correlation. *p<0.05 and ***p<0.001. Abbreviations: controls, disease controls; DDC, 3,5-diethoxycarbonyl-1,4-dihydrocollidine; fibro1, fibrosis1; fibro2, fibrosis2; pare, parenchyma; PSC, primary sclerosing cholangitis.

As several analytical approaches showed robust inflammatory signals adjacent to high metallothionein expression, we asked if biliary inflammation correlates with metallothionein expression in an animal model of PSC. To induce biliary damage, wild-type C57BL/6J mice were fed a 0.1% diet of DDC for 14–28 days,^29^ followed by quantification of metallothionein 1 (*Mt1*) upregulation (Figure 6A). We detected significantly higher levels of *Mt1* expression (*p*=0.03) in DDC-fed animals (n=5) compared to control mice receiving standard chow (n=7), directly demonstrating that biliary inflammation associates with increased metallothionein expression in a murine model of PSC (Figure 6B).

Having shown that biliary inflammation induces *Mt1* expression in mice, we lastly asked if metallothionein activity correlated with clinical parameters in patients. Using fractions of *MT1G*+ parenchyma as a measure of metallothionein activity (Figure 6C), we observed that metallothionein expression positively correlated with several biochemical metrics of liver disease at time of transplantation (Figure 6D and Supplemental Table S1, http://links.lww.com/HEP/J842), including AST (*R*=0.45, *p*=0.013), ALT (*R*=0.48, *p*=0.007), ALP (*R*=0.54, *p*=0.002), and bilirubin (*R*=0.46, *p*=0.01).

## DISCUSSION

The uneven distribution of the liver pathology, even at the microanatomical level, and the progressive development of fibrosis in PSC are major hurdles for understanding localized disease mechanisms and the discovery of novel therapeutic targets. Previous studies have identified disease-associated immune^6–9,11–16,30^ and fibrosis signatures,^14,30^ but restricted to traditional transcriptomic methodologies lacking spatial granularity or have been performed on sorted cells, which is biased by the preselection of sorting markers and prohibits the direct spatial assessment of local gene pathways and cell types within the same specimen. To circumvent these limitations, we combined spatial transcriptomics with patient-paired snRNA-seq and examined the largest number of PSC explants to date using microanatomical classification of different liver regions.

Using a larger number of PSC and disease controls compared to previous spatial RNA studies,^15,16^ we identified a robust metallothionein signature within the parenchyma–fibrosis interface in PSC livers by spatial transcriptomics and showed using snRNA-seq that the strongest metallothionein signals originated from hep.edge hepatocytes. Localization of metallothionein-positive hep.edge hepatocytes by spatial transcriptomics confirmed their close proximity to fibrosis by IHC protein staining. The relevance of this pathway for bile duct inflammation was validated in the DDC mouse model of PSC, where we observed heightened metallothionein expression following DDC-induced biliary inflammation. In addition to these novel findings, our spatial sequencing of microanatomical regions along with snRNA-seq data highlighted several regions and cell types that were also reported by Andrews et al,^16^ namely the presence of cholangiocyte-like hepatocytes (hep.edge) localized to the perimeter of PSC scars in direct contact with parenchyma regions defined by spatial transcriptomics.

Metallothioneins are ubiquitous intracellular cysteine-rich proteins that maintain intracellular homeostasis of metal ions and are induced by a diverse range of factors released upon oxidative stress and acute-phase immune reactions, including transition metals, proinflammatory cytokines, superoxides, hydroxyl anions, and steroids.^31^ Previous findings in a descriptive study by Alscher et al.^32^ detected metallothionein in liver specimens from a range of etiologies, with the highest metallothionein expression in proinflammatory and cholestatic conditions consistent with typical features of PSC. Besides the aforementioned study, metallothionein has not been highlighted previously in PSC, and our study is the first to report a robust upregulation of metallothionein in PSC localized to specific microanatomical regions and provide functional evidence in a mouse model of PSC. Metallothionein activation in PSC livers could be related to the inflamed gut–liver axis, dysbiosis^33,34^ or increased intestinal permeability^35,36^ as Lahiri and Abraham^37^ demonstrated that chronic stimulation of pattern recognition receptors on macrophages increases levels of intracellular metallothioneins and enhances bacteria clearance. Though we observed upregulation of the pathway in a PSC mouse model, further mechanistic studies dedicated to uncovering the central elements of the metallothionein pathway during bile duct inflammation, which can be pharmacologically targeted, are necessary. DEG analysis of *metallo* ^high^ and *metallo* ^low^ regions defined as parenchyma or fibrosis by spatial transcriptomics demonstrated few DEGs in the parenchyma and a high number in fibrotic regions. As expected, pathway enrichment analysis identified the metallothionein pathway, but those relating to bile secretion, bile acid biosynthesis, and PPARγ signaling were also elevated in fibrotic *metallo* ^high^ regions. Speculatively, these findings imply that cholestasis in PSC could induce metallothionein expression and drive dysregulated energy, amino acid, and lipid metabolism in *metallo* ^high^ liver regions.

Since PSC affects the liver unevenly, we initially hypothesized that the driving factors for disease differed between the central region and periphery and that stronger disease signatures would be observed in the central regions. Instead, our data suggested that gene profiles of central and peripheral liver biopsies were completely comparable within the same microanatomical regions as classified by spatial transcriptomics (pare, fibro1, fibro2). This implies that major disease processes are consistent irrespective of sampling location and that potential targetable pathways would be the same. Importantly, it should be acknowledged that the lack of striking disease signatures between central and peripheral biopsies may also reflect imprecise sampling just after explantation of the diseased liver. Recent advancements in the spatial analysis of FFPE tissue^38^ will allow direct assessment of the exact same tissue scored by clinical pathologists prior to spatial analysis, and the topic of differing disease pathways in microanatomical regions of the PSC liver should be assessed in the future by these approaches. Despite the inclusion of central and peripheral liver biopsies, our analyses were undoubtedly still restricted to small portions of the explanted liver, but the observations of similar disease pathways in central and peripheral regions increase the generalizability of the findings. We also compared the spatial gene content of each parenchyma and fibrosis subcluster between individuals transplanted at early-stage and late-stage PSC and noted that phenotypic and mechanistic processes within specific subregions of fibrosis (fibro1.3, fibro2.2) harbor the greatest differences, though our conclusions are drawn on the analysis of few early and end-stage patients. It is also important to note that our overall study size remains relatively small, and further validation in larger cohorts is warranted.

In conclusion, we have demonstrated the utility of applying combinatorial spatial and high-resolution single-cell methodologies to study complex disease networks in PSC and uncovered metallothionein as a novel pathway that could guide further studies aiming to develop effective medical treatments for PSC.

## Supplementary Material
