# Supplementary material for: Multimodal transcriptomics identifies metallothionein as a novel pathway in primary sclerosing cholangitis
Source: Hepatology. 2025 Jun 25;83(5):1066–81. doi: 10.1097/HEP.0000000000001432 (PMC13089832; doi:10.1097/HEP.0000000000001432)
Supplement: Supplementary file 1 [file hep-83-1066-s001.pdf]

Multimodal transcriptomics identifies metallothionein as a novel pathway in primary sclerosing cholangitis

Brian K. Chung*, Markus S. Jördens*, Jonas Øgaard, Henrik Mikael Reims, Benedicte Flateland, Tom Luedde, Xiaojun Jiang, Tom H. Karlsen and Espen Melum

* Equally contributing first authors

Table of Contents

Supplementary Materials & Methods 2

Supplementary References 5

Supplementary Figures 6

Supplementary Tables 17

Supplementary Materials & Methods

Tissue processing and nuclei isolation

Tissue for snRNA-seq was prepared using Chromium nuclei isolation kits with RNase inhibitor (10x Genomics, Pleasanton, CA, USA) according to the manufacturer's recommendations (Rev A, 10x Genomics). Approximately 25 mg of liver tissue was placed into 10X lysis buffer and processed into a single-cell suspension using a mortar and pestle. Nuclei were isolated using Chromium nuclei isolation columns and were purified by centrifugation using debris removal buffer. Isolated nuclei were resuspended in wash and resuspension buffer and counted by Hoechst staining using a fluorescence microscope.

Spatial transcriptomics

Frozen OCT-embedded explant tissue from central and peripheral liver locations were sectioned at 10 µm and placed on Visium RNA capture slides for spatial transcriptomics according to the manufacturer's recommendations (10x Genomics). Liver tissues were mounted on RNA capture slides and heated to 37°C for 1 min, fixed in methanol at -20°C for 30 min and stained with aqueous hematoxylin for 7 min and eosin for 1 min. HE slides were imaged using an Axio Scan.Z1 slide scanner (ZEISS, Oberkochen, Germany) and placed in Visium slide cassettes for *in situ* polyadenylation using yeast poly(A) polymerase (yPAP; Thermo Fisher Scientific, Waltham, MD, USA.[1] Slides were equilibrated for 30 s at RT using wash buffer (5X yPAP reaction buffer, 40 U/µL Protector RNase inhibitor; Roche, Basel, Switzerland). After equilibration, buffer was removed and yPAP enzyme mixture (5X yPAP reaction buffer, 600 U/µL yPAP enzyme, 25 mM ATP, Protector RNase Inhibitor (40 U/µL) was added to each slide for 25 mins at 37°C. Slides were washed and then incubated in standard Visium permeabilization buffer for 27 mins. Optimal incubation period was determined empirically using Visium optimization kits (10x Genomics) in accordance with the manufacturer's protocol (Rev E; 10x Genomics). After permeabilization, slides were processed for reverse transcription, second-strand synthesis, denaturation and cDNA amplification. Expected product size and sufficient product yield was verified using High Sensitivity DNA assays (Agilent Technologies, Santa Clara, CA, USA) run on an Agilent 2100 Bioanalyzer. Sequencing depth was adjusted to achieve a minimum of 50,000 reads per RNA capture spot.

Single nuclei RNA sequencing

SnRNA-seq was performed using Chromium Next GEM single cell 3' kits (v3.1, 10x Genomics) following the manufacture's protocol (Rev E, 10x Genomics). 30,000 nuclei per reaction (target recovery of 10,000 nuclei) were loaded into ChipG chips and processed on a Chromium iX (10x Genomics) to generate nuclei gel bead emulsions (GEMs). SnRNA-seq libraries were prepared using GEM reverse transcription (GEM-RT) on a Veriti 96-well thermal cycler (Applied Biosystems, Foster City, CA, USA) and cDNA was purified using DynaBeads MyOne silane beads (Thermo Fisher Scientific) and SPRIselect reagent kit (Beckman Coulter, Brea, CA, USA). cDNA was amplified using a 96-well thermocycler set to 98°C for 3 min; 12 cycles: 98°C for 15 s, 67°C for 20 s and 72°C for 1 min; 72°C for 1 min; end at 4°C. Amplified cDNA was purified, fragmented and indexed into sequencing libraries by end repair, A-tailing, adapter

ligation, SPRIselect cleanup and sample index PCR. Sequencing depth was adjusted to achieve a minimum of 25,000 reads per nuclei.

Sequencing and pre-processing of spatial and snRNA-seq transcriptomics

Paired-end, dual-index sequencing was performed on a Novaseq (Illumina, San Diego, CA, USA) S4 ¼ flow cell (2-2.5 billion reads per end, 300 cycles) following the manufacture's recommend settings (10x Genomics). Sequencing reads were demultiplexed and aligned to human genome GRCh39 using Space Ranger v.1.1.0 (10x Genomics) for spatial transcriptomics and Cell Ranger v.1.1.0 (10x Genomics) for snRNA-seq. All RNA capture spots with gene content were automatically aligned to the corresponding hematoxylin and eosin (HE) image using the default image registration algorithm in Space Ranger. Nuclei with ambient mitochondrial gene content greater than 5% of the total unique molecular identifier (UMI) gene count were excluded.

Pseudobulk, pathway enrichment analysis and CIBERSORTx gene deconvolution

DEGs identified by Pseudobulk were analyzed for pathway enrichment using Enrichr[2-4] and the Kyoto Encyclopedia of Genes and Genomes database (KEGG).[5-7] Enriched pathways in PSC and disease controls reached statistical significance (P<0.01) using the Fisher's exact test.

SnRNA-seq profiles for 9 major cell types identified in 12 PSC and 4 disease control explants also assessed by spatial transcriptomics were converted into disease-specific signature matrix references using CIBERSORTx.[8] To estimate cell fractions in metallothionein (*metallo*) regions, log-normalized sum expression values of *MT1G* and *MT1H* measured by spatial transcriptomics were divided into tertiles (Q1-Q3) and RNA capture spots corresponding to Q1 ($metallo^{low}$), Q2 ($metallo^{inter}$) and Q3 ($metallo^{high}$) were analyzed in Loupe browser. Normalized log2 fold-change values of all significant DEGs (P<0.01) from Q1, Q2 and Q3 were compiled into CIBERSORTx mixture files. Cell fractions within Q1, Q2 and Q3 were estimated by CIBERSORTx using the relevant mixture file and disease-specific signature matrix reference. Batch and quantile normalization disabled, run mode set to relative and permutations set to 1000.

Immunohistochemistry and QuPATHstaining quantification

Formalin-fixed paraffin-embedded (FFPE) human liver samples from PSC and cirrhotic controls were sectioned at 3 µm and fixed to Superfrost Plus microscope slides (Thermo Fisher Scientific) at 60°C for 3 hr. Slides were deparaffinized using Histolab-Clear (Histolab, Askim, Sweden), and rehydrated and boiled at 95°C for 20 min in citrate buffer (10 mM sodium citrate, 0.05 % Tween20, pH 6.0) for antigen retrieval. Endogenous peroxidase activity was blocked using 3% H_2O_2 for 20 min. Slides were washed 3x for 5 min in 1% PBS-Tween20 followed by blocking for 60 min at RT using SuperBlock T20 (PBS) blocking buffer (Thermo Fisher Scientific). Blocking buffer was discarded and tissues were immediately stained overnight at 4°C with rabbit anti-human MT1G polyclonal (PA5-144126, Thermo Fisher Scientific), rabbit anti-human APOE antibody (EPR19392, Abcam, Cambridge, UK) or rabbit anti-human KRT19 antibody (A53-B, Abcam) at a 1:250 dilution of PBS with 0.25% horse serum, 1% BSA, 10% SuperBlock T20 and 0.05%

Tween20. Slides stained with primary antibody were washed 3x in PBS-T and incubated with 3 drops of ImmPRESS® HRP horse anti-rabbit IgG peroxidase (Vector Laboratories, Newark, CA, USA) for 30 min at RT. Slides were washed 3x to remove primary antibodies and 150 µl of ImmPACT® DAB peroxidase (Vector Laboratories) was added per slide for 30 s (KRT19), 60 s (APOE) or 180 s (MT1G). Reactions were quenched with water and slides were counterstained using hematoxylin QS (Vector Laboratories) for 15 s. Slides were again washed with water, dehydrated in ethanol and cleared in Histolab-Clear (Histolab) before mounting with Eukitt quick-hardening mounting medium (Sigma-Aldrich, St. Louis, MO, USA) and glass coverslips. Slides were imaged using an Axio Scan.Z1 (ZEISS) microscope and imaged using a HV-F202SCL (Hitachi, Tokyo, Japan) slide scanner equipped with a TL LED lamp. KRT19-stained IHC samples (n=27) were analyzed using QuPath.[9] Cells were detected using the deep learning based StartDist plugin[10] and a random forest classification model was trained to differentiate positively stained cholangiocytes from other cells in a reiterative process until no erroneously predicted cholangiocytes were identified across a subset of the samples (n=10). Cholangiocyte fractions as a ratio of all cells were then calculated for each sample.

Supplementary References

1. McKellar DW, Mantri M, Hinchman MM, et al. Spatial mapping of the total transcriptome by in situ polyadenylation. Nat Biotechnol 2023;41:513-520.

2. Chen EY, Tan CM, Kou Y, et al. Enrichr: interactive and collaborative HTML5 gene list enrichment analysis tool. BMC Bioinformatics 2013;14:128.

3. Kuleshov MV, Jones MR, Rouillard AD, et al. Enrichr: a comprehensive gene set enrichment analysis web server 2016 update. Nucleic Acids Res 2016;44:W90-7.

4. Xie Z, Bailey A, Kuleshov MV, et al. Gene Set Knowledge Discovery with Enrichr. Curr Protoc 2021;1:e90.

5. Kanehisa M, Goto S. KEGG: kyoto encyclopedia of genes and genomes. Nucleic Acids Res 2000;28:27-30.

6. Kanehisa M. Toward understanding the origin and evolution of cellular organisms. Protein Sci 2019;28:1947-1951.

7. Kanehisa M, Furumichi M, Sato Y, et al. KEGG for taxonomy-based analysis of pathways and genomes. Nucleic Acids Res 2023;51:D587-D592.

8. Newman AM, Steen CB, Liu CL, et al. Determining cell type abundance and expression from bulk tissues with digital cytometry. Nat Biotechnol 2019;37:773-782.

9. Bankhead P, Loughrey MB, Fernandez JA, et al. QuPath: Open source software for digital pathology image analysis. Sci Rep 2017;7:16878.

10. Weigert M, Schmidt U. Nuclei Instance Segmentation and Classification in Histopathology Images with Stardist. 2022 IEEE International Symposium on Biomedical Imaging Challenges (ISBIC). Kolkata, India: IEEE, 2022:pp. 1-4.

Supplementary Figures

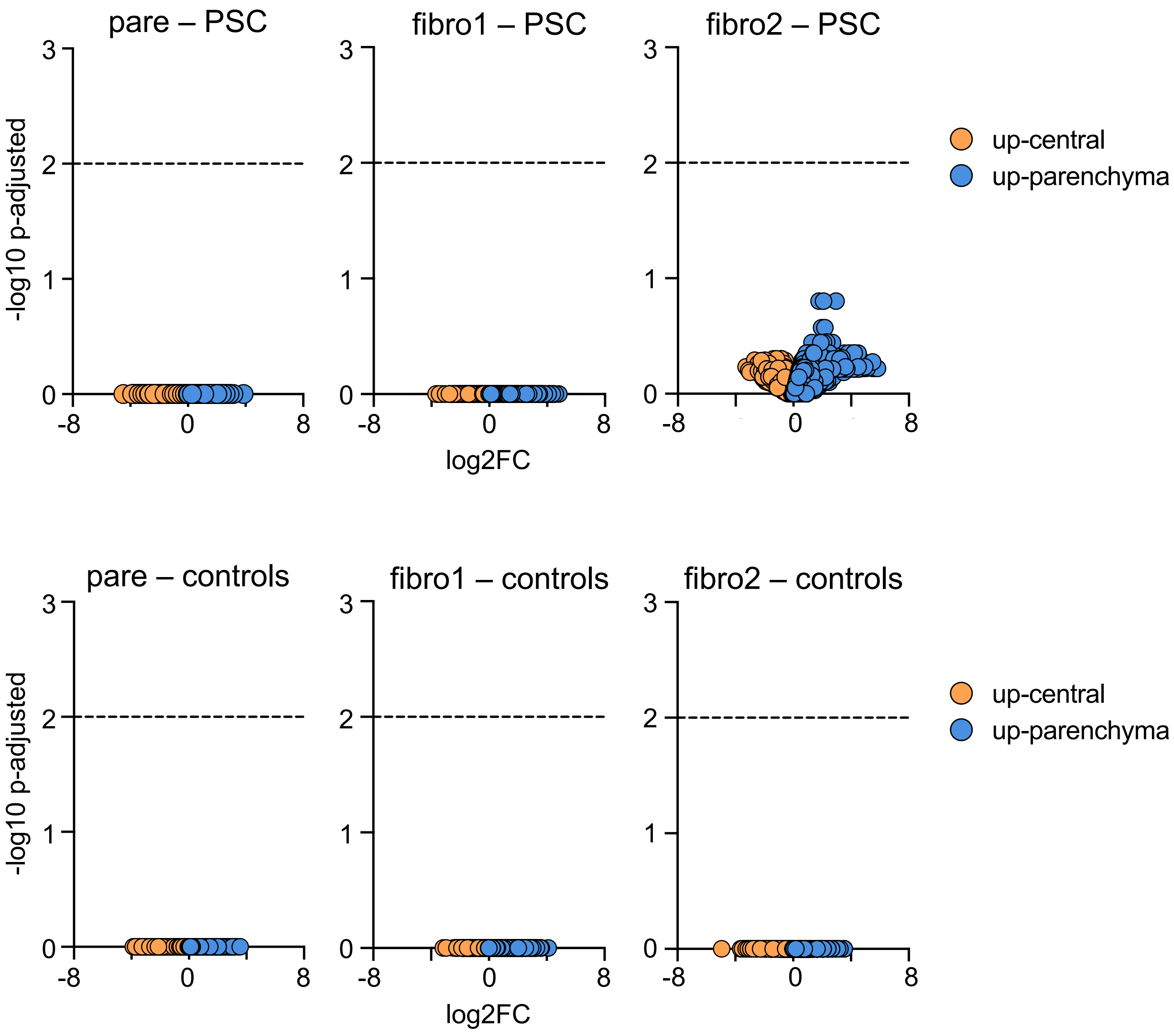

Supplementary Figure 1. Differential gene expression analysis of micro-anatomical liver regions in central and peripheral biopsies from PSC and disease control explants. Gene content of micro-anatomical liver regions defined by spatial transcriptomics compared in patient-matched central and peripheral liver biopsies from PSC (n=23) and disease control explants (n=7) using the exact negative binomial test by Pseudobulk analysis. Pare, parenchyma; fibro1, fibrosis1; fibro2, fibrosis2; log2FC, log2 fold-change.

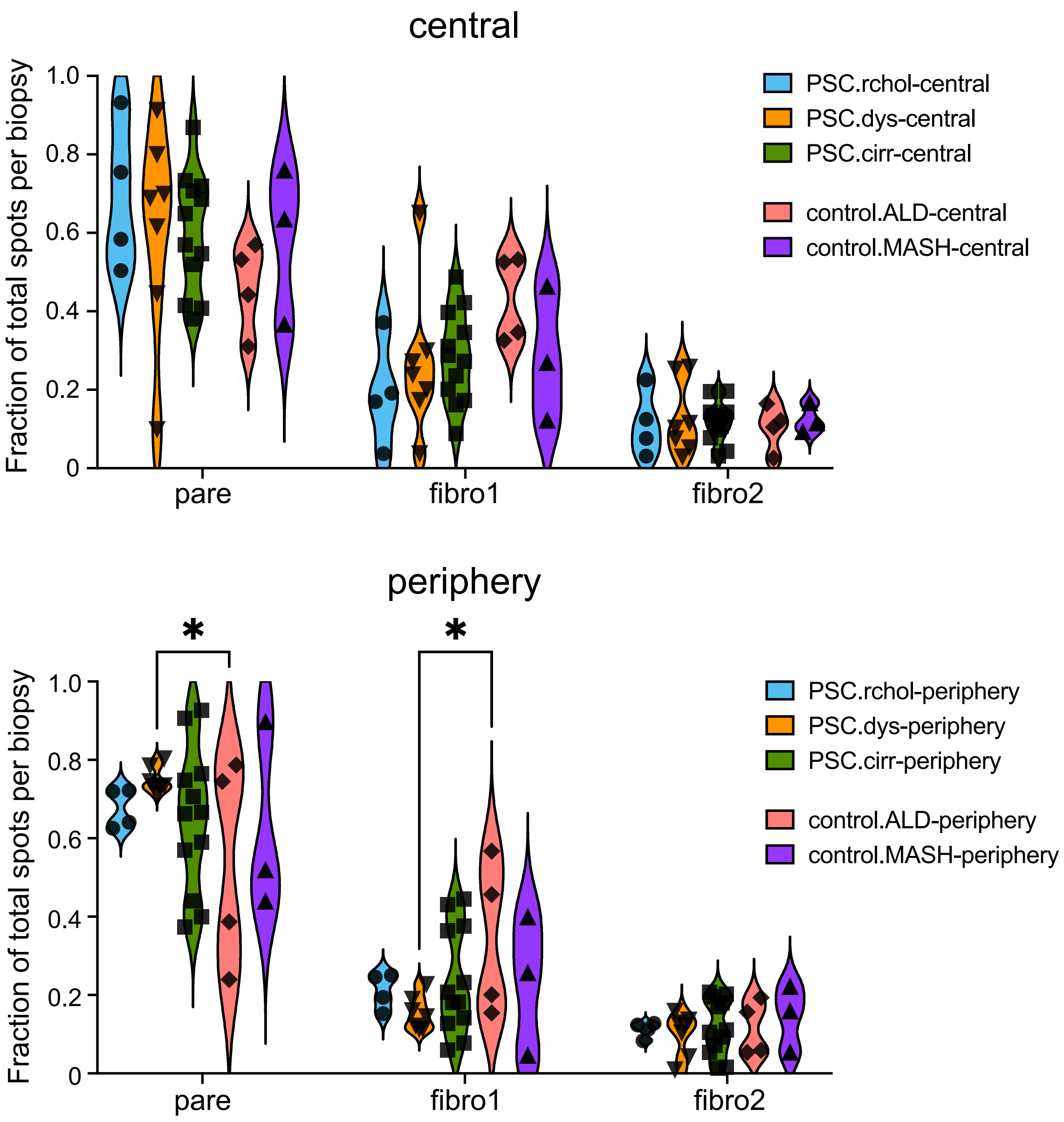

Supplementary Figure 2. Enumeration of micro-anatomical liver regions in subgroups of PSC and disease control explants. Proportion of parenchyma (pare), fibrosis1 (fibro1) and fibrosis2 (fibro2) RNA capture spots enumerated as a fraction of total spots per central or peripheral liver biopsy assessed by spatial transcriptomics. Statistical significance evaluated by two-way ANOVA. *P<0.05. PSC, primary sclerosing cholangitis; ALD, alcoholic-related liver disease; MASH, metabolic dysfunction-associated steatohepatitis; rchol, recurrent cholangitis; dys, dysplasia; cirr, cirrhosis.

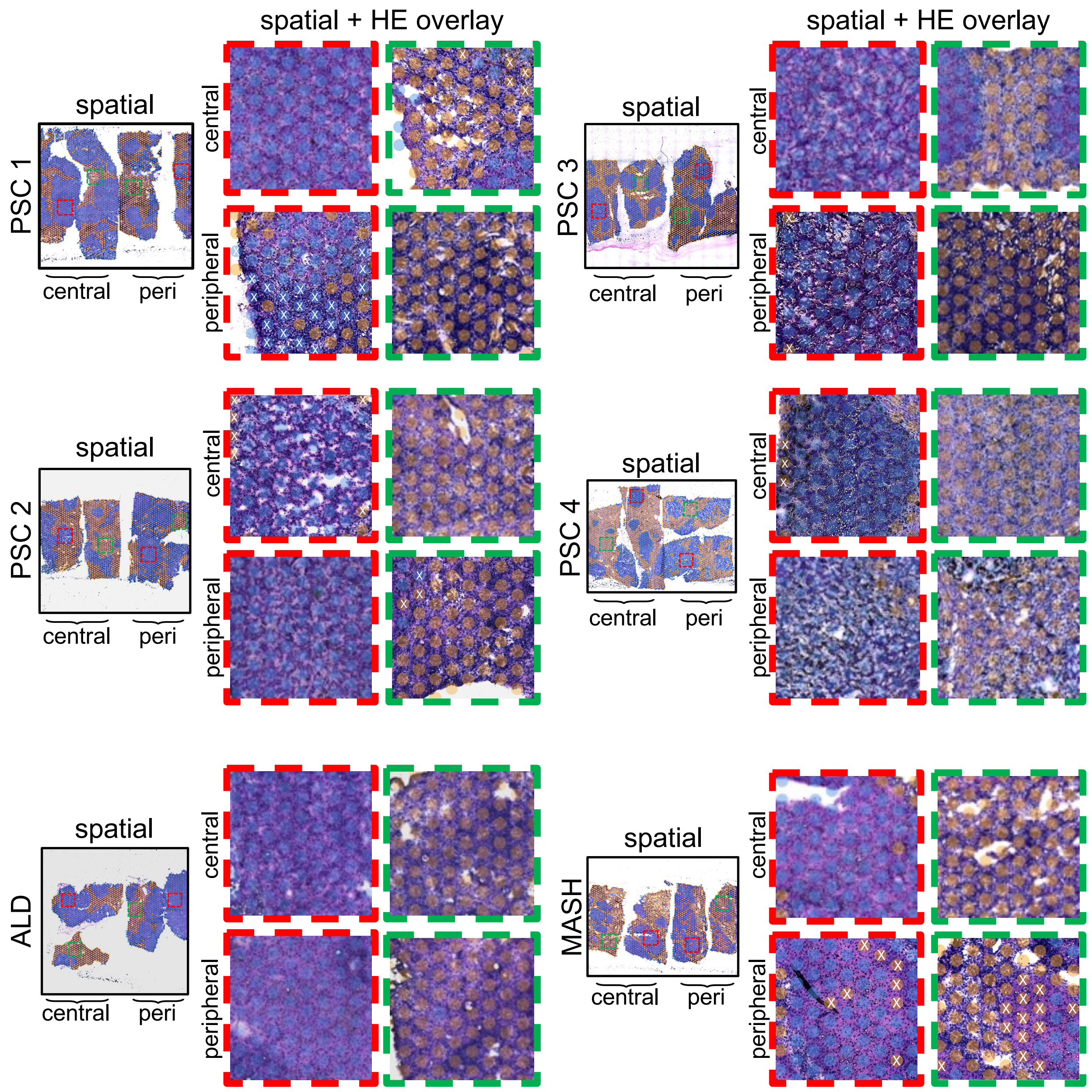

Supplementary Figure 3. Representative hematoxylin and eosin (HE) staining overlaid with spatial transcriptomes of central and peripheral liver tissue from primary sclerosing cholangitis (PSC, n=4) and non-PSC disease controls (ALD, n=1 and MASH, n=1). Regions classified by spatial transcriptomics as parenchyma (red squares, blue spots) or fibrosis (green squares, orange spots) were assessed. Spots labeled with 'X' represent disconcordant classification between spatial transcriptomics and an expert liver pathologist (HMR) and all unmarked spots had concordant classification.

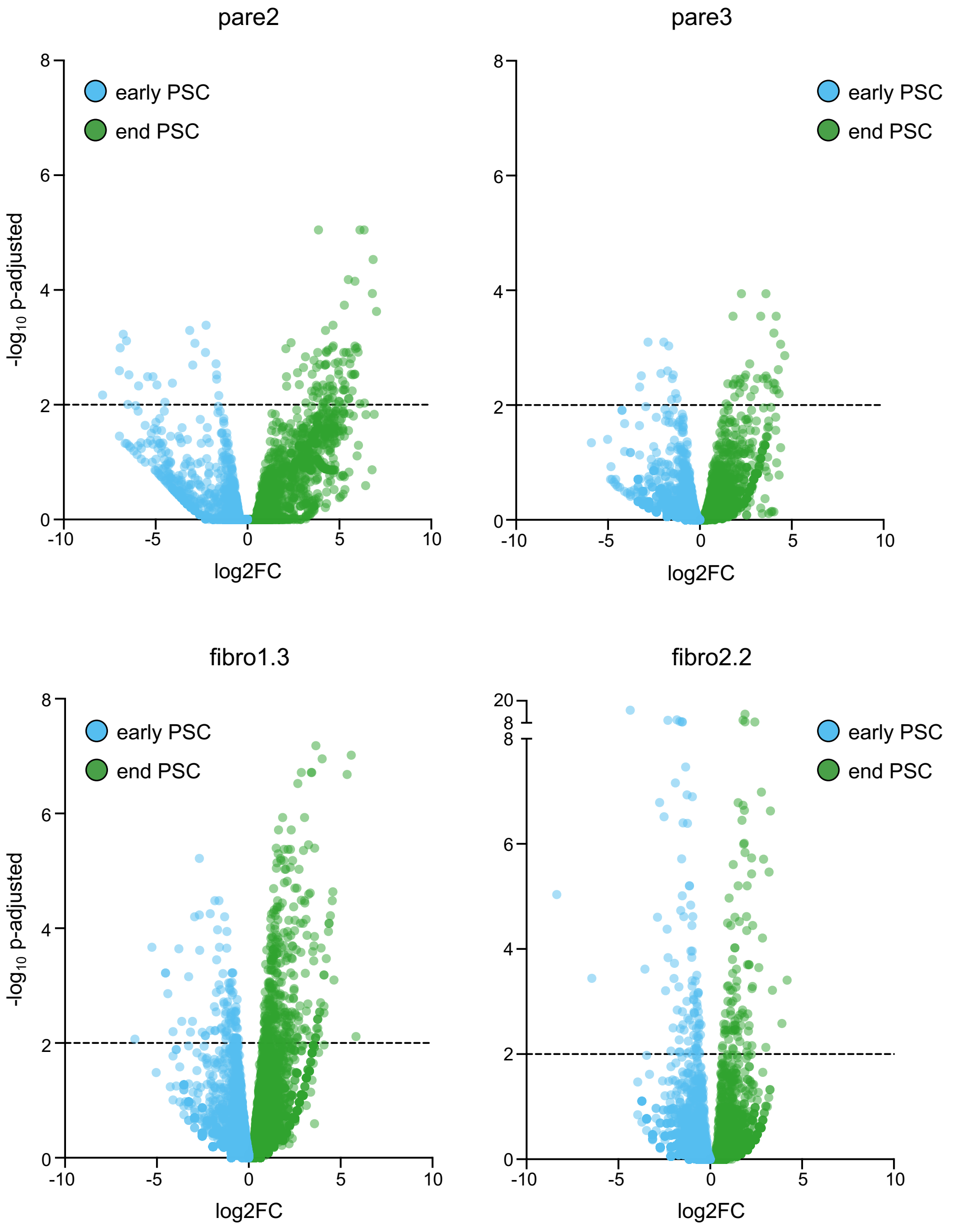

Supplementary Figure 4. DEG analysis of parenchyma and fibrosis subcluster regions defined by spatial transcriptomics in early-stage and end-stage PSC explants. Dotted line represents P-adjusted=0.01. Statistical significance evaluated using exact negative binomial test. Early: early-stage, end: end-stage, FC: fold-change.

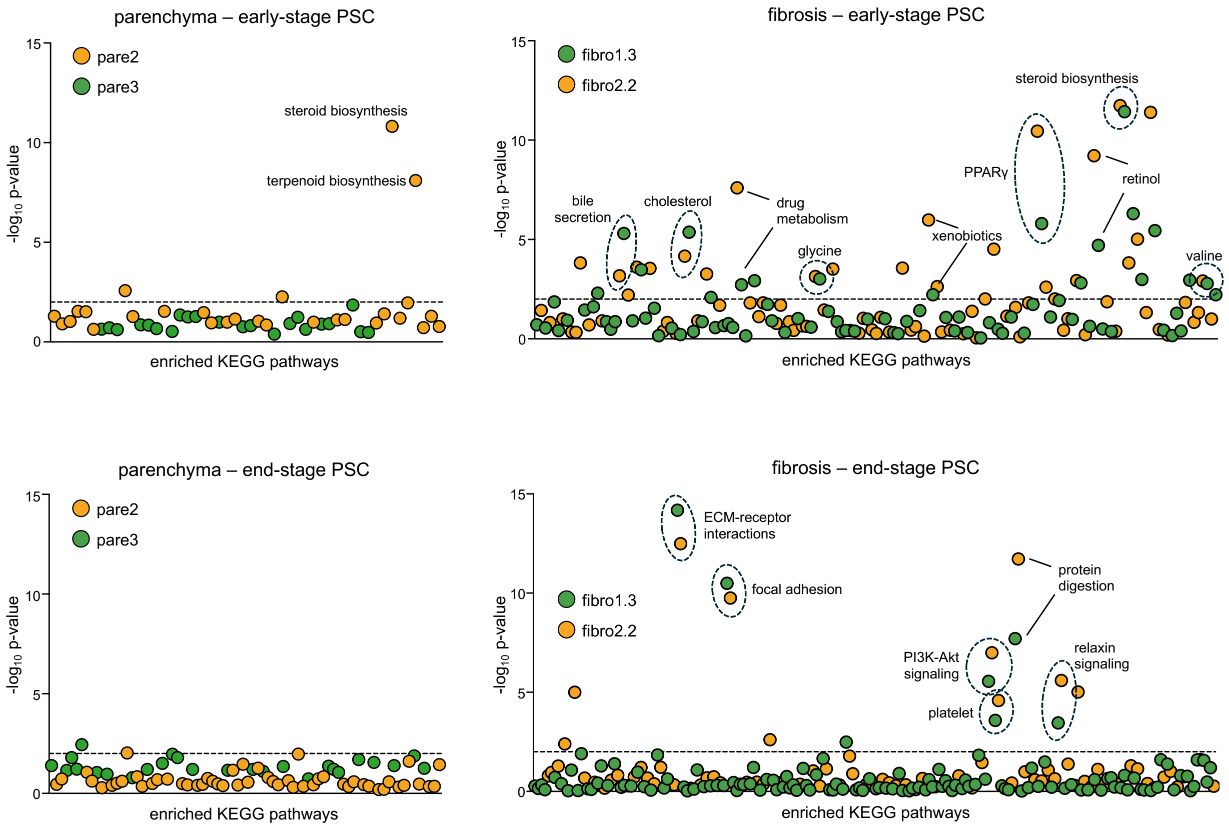

Supplementary Figure 5. KEGG pathway enrichment analysis of DEGs detected in subregions of parenchyma (pare2, pare3) and fibrosis (fibro1.3, fibro2.2) as defined by spatial transcriptomics. Dotted lines represent P=0.01 Statistical enriched pathways evaluated by Fisher's exact test. Cholesterol: cholesterol metabolism Glycine: glycine, serine and threonine metabolism, platelet: platelet activation, valine: valine, leucine and isoleucine degradation.

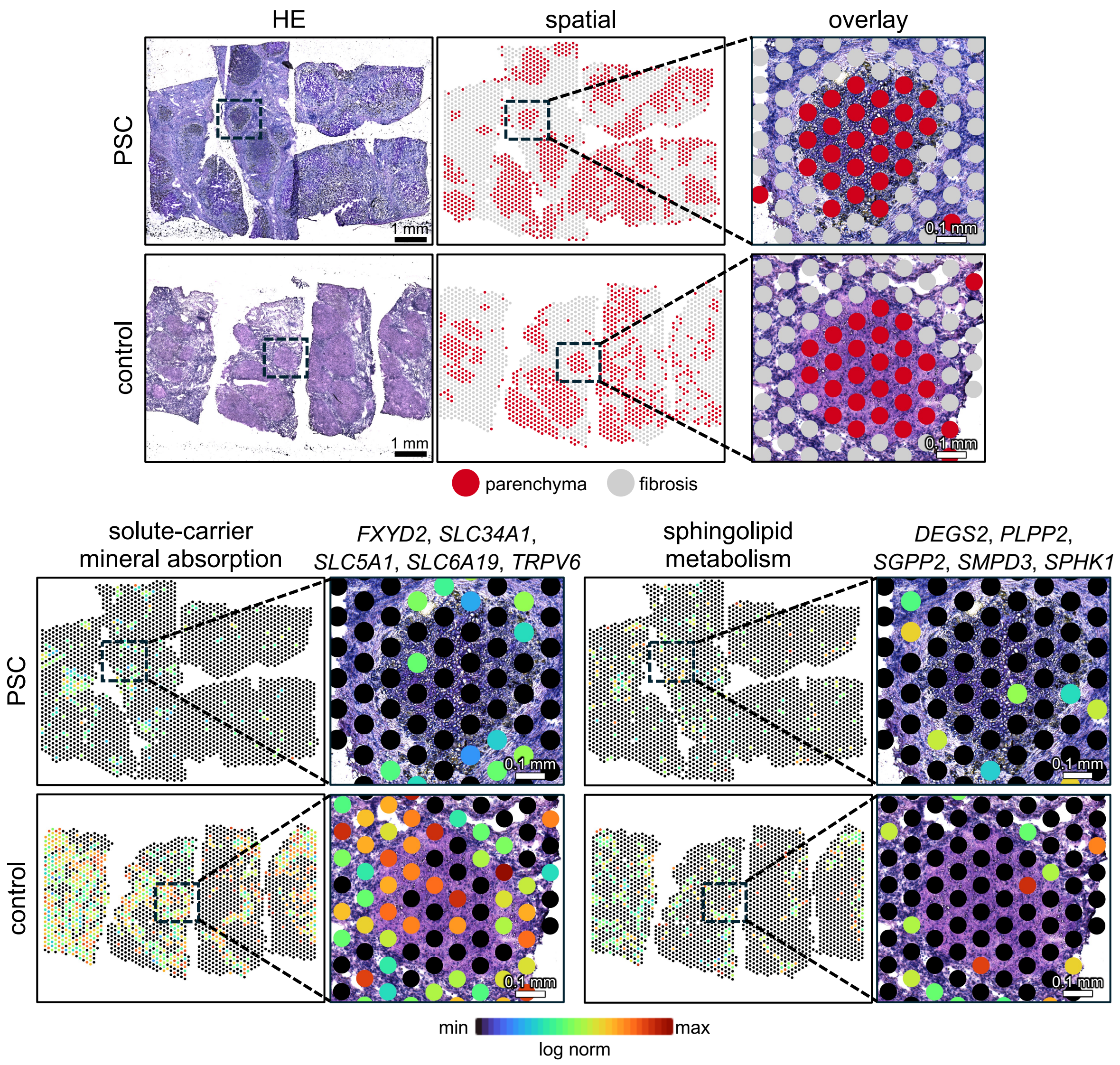

Supplementary Figure 6. Solute mineral absorption and sphingolipid metabolism pathways are enriched in the parenchyma of disease control livers. Representative images of parenchyma (red) and fibrosis (grey) as defined by spatial transcriptomics. Dashed boxes show magnified regions of hematoxylin and eosin (HE) stainings and spatial transcriptomics of solute mineral absorption and sphingolipid metabolism markers. Log-normalized expression sum values of indicated genes measured by spatial transcriptomics are overlaid onto corresponding magnified HE images.

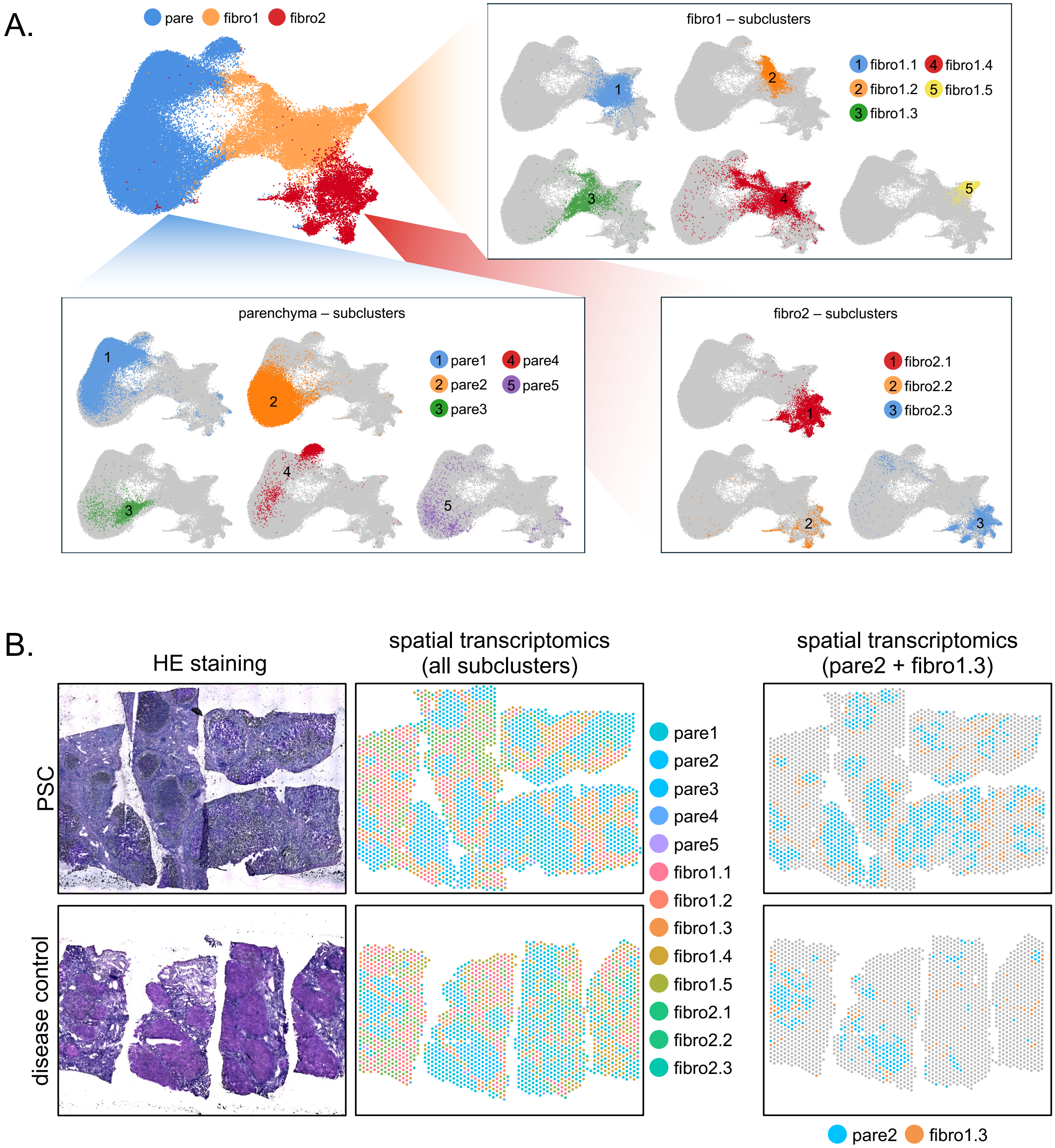

Supplementary Figure 7. UMAP and tissue localization of spatial transcriptomic subclusters. (A) Spatial transcriptomic UMAPs of entire study cohort (n=30) split by subclusters. (B) Representative hematoxylin and eosin (HE) stainings and spatial plots of PSC and disease control tissue showing all 13 spatial subclusters (pare1-5, fibro1.1-1.5, fibro2.1-2.3) and pare2 and fibro1.3 alone.

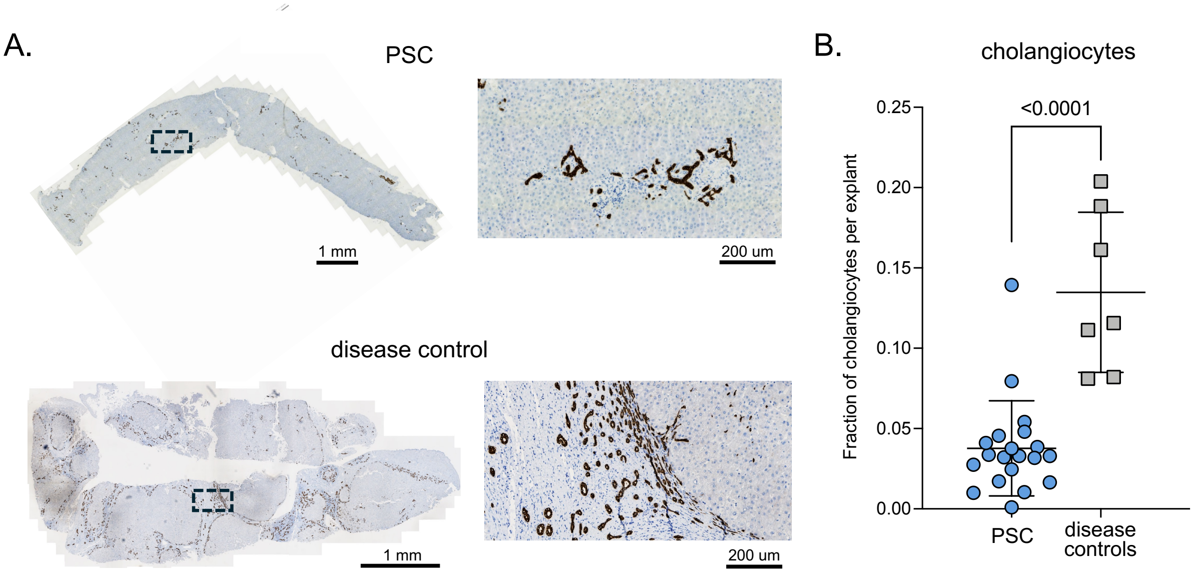

Supplementary Figure 8. Fraction of cholangiocytes as measured by KRT19 staining by immunohistochemistry (IHC) of 20 PSC and 7 disease control explants. (A) Representative tissue scans and magnified images of KRT19 IHC tissue staining from PSC and disease control explant. (B) Mean fraction of cholangiocytes normalized to total number of cells per explant as measured by IHC and QuPATH analysis. Error bars indicate standard deviation, statistical significance evaluated using Mann-Whitney.

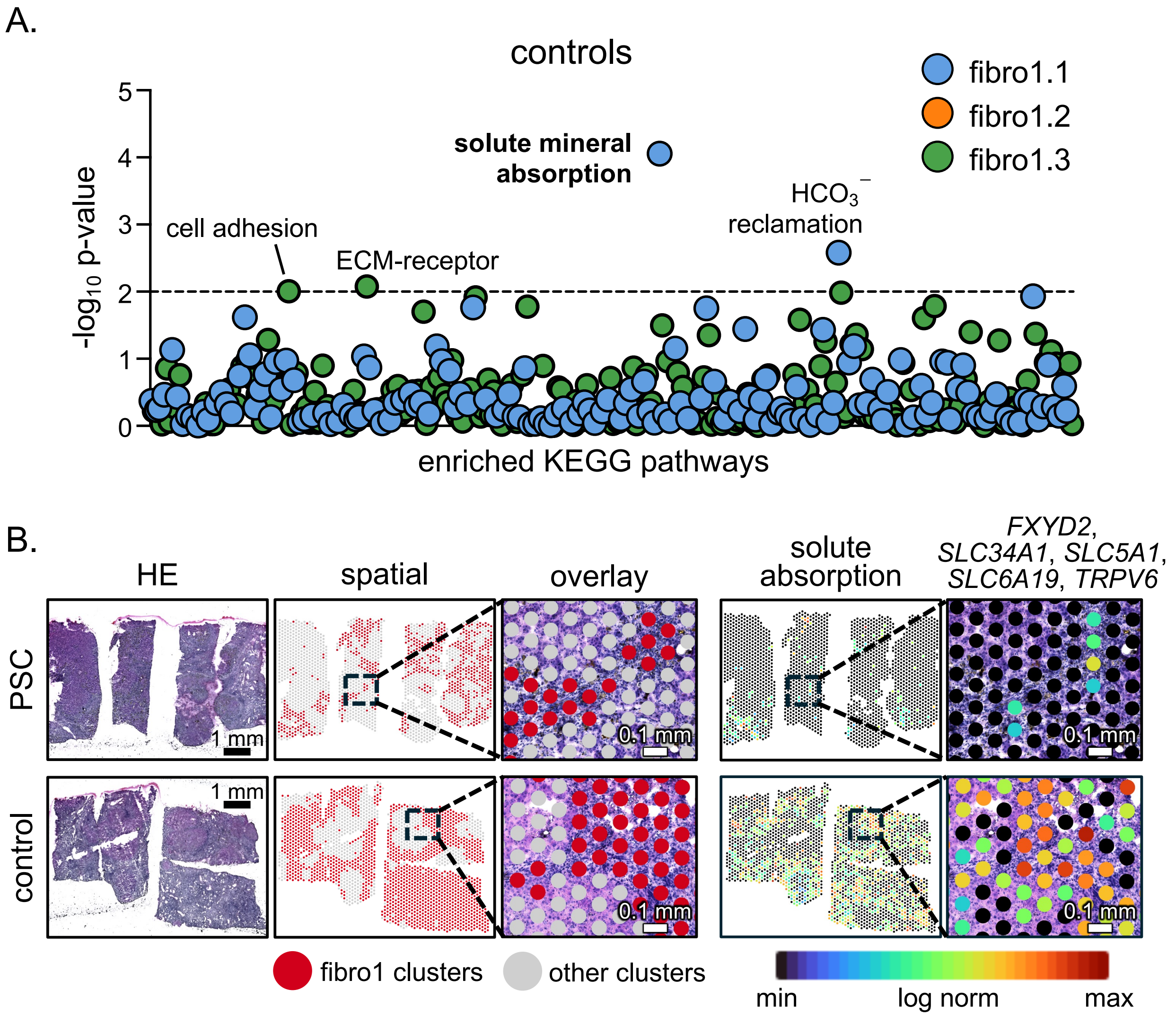

Supplementary Figure 9. Solute mineral absorption is enriched in fibrosis1 (fibro1) of control liver explants. (A) Pathway enrichment analysis of differentially-expressed genes in fibro1 identifies solute mineral absorption as the most significantly overrepresented pathway in disease controls versus PSC. (B) Representative spatial mapping of fibro1 overlaid onto magnified hematoxylin and eosin (HE) liver images from PSC and disease controls. Log-normalized gene expression sum of solute mineral absorption markers (*FXYD2*, *SLC34A1*, *SLC5A1*, *SLC6A19*, *TRPV6*) shown for the entire liver section and also superimposed over magnified HE areas outlined in dotted boxes.

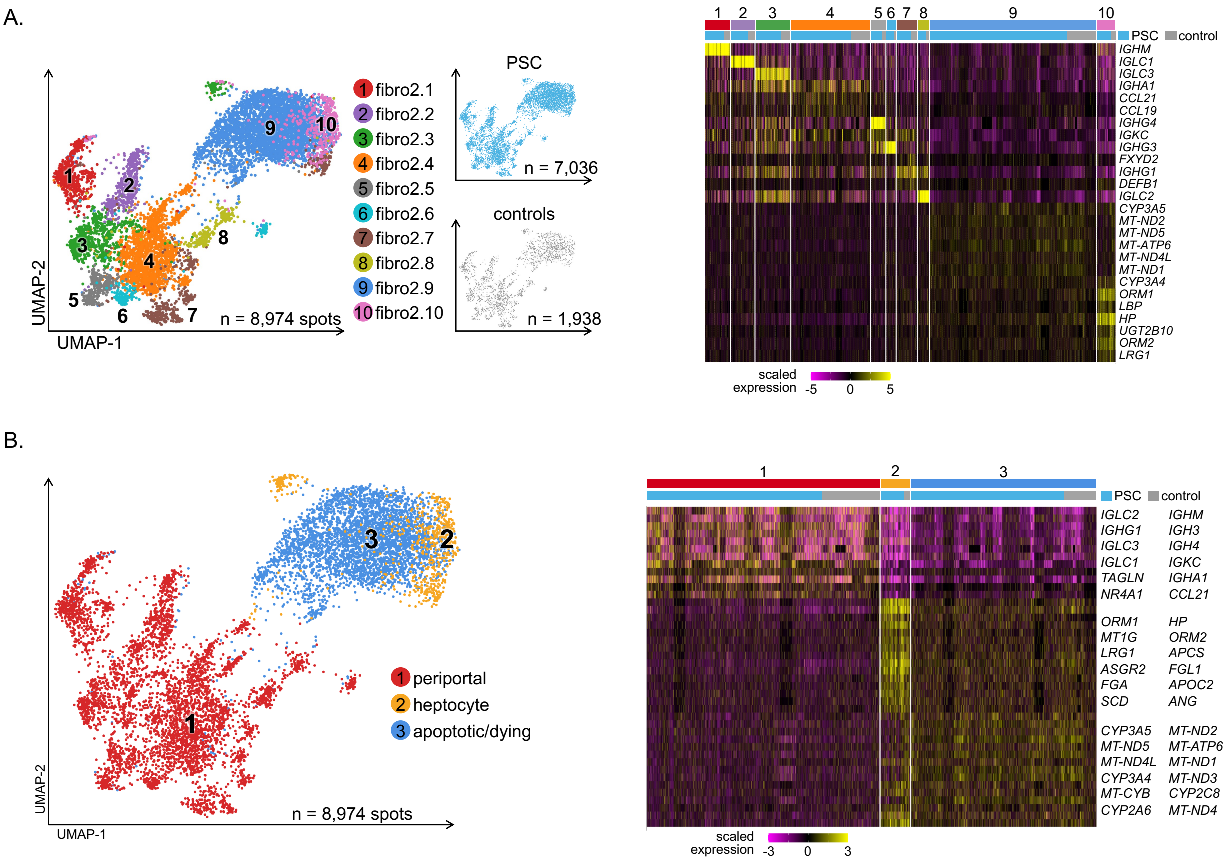

Supplementary Figure 10. Spatial transcriptomics of fibro2 regions defined by high immunoglobulin (Ig) content shows B cells and plasma cells primarily localize to periportal niches in PSC and disease control livers (A) UMAP of 8,974 fibro2 RNA capture spots grouped by cluster analysis of spatial transcriptomes and split into PSC and disease controls. Heatmap shows differential expressed genes in each cluster. (B) UMAP of same fibro2 spots after merging periportal, hepatocyte and apoptotic/dying cell clusters. Heatmap shows top 12 differentially expressed genes in each cluster.

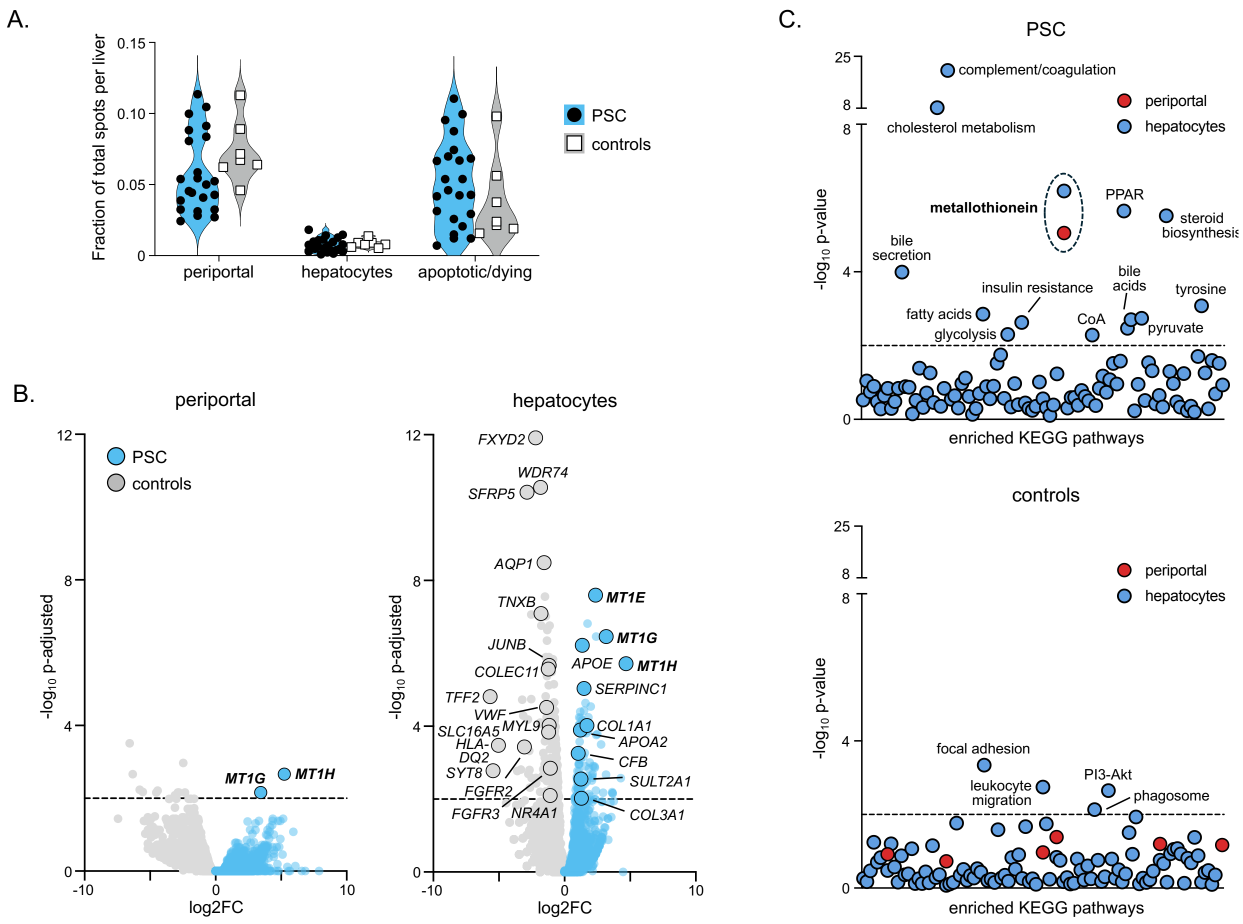

Supplementary Figure 11. Differential gene expression and pathway enrichment of fibro2 closely resembles changes observed in comparison of fibro1 between PSC and disease controls. (A) Relative abundance of spots classified into fibro2 subclusters by spatial transcriptomics. Cluster fractions represent the number of RNA captures spots per fibro2 subcluster as a proportion of total liver spots. (B) Differential gene analysis of periportal and hepatocyte subclusters in fibro2 from PSC and controls. (C) Pathway enrichment analysis of DEG content from fibro2 periportal and hepatocyte subclusters of PSC and controls. Statistical significance evaluated for (A) using Mann-Whitney U-test and unpaired Student's t-test, for (B) using exact negative binomial test and for (C) using Fisher's exact test. Dotted lines in (B) and (C) indicate P-adjusted=0.01 and P=0.01.

Supplementary Tables

Supplementary Table 1. Clinical details of study cohort at time of liver transplantation

Patient ID	Disease	Transplant indication	Age (years)	Sex (M/F)	IBD status	UDCA (Y/N)	APRI	FIB-4	MELD Na	Hb (g/dL)	Erythro (10^{12}/L)	Platelets (10^{9}/L)	Leuko (10^{9}/L)	INR	Sodium (mmol/L)	AST (U/L)	ALT (U/L)
1	PSC	dysplasia	56	M	neg	N	0.2	0.72	6	13.4	4.4	366	8.5	1	140	43	84
2	PSC	dysplasia	41	M	UC	N	0.8	1.44	6	13.4	4.4	287	8.9	1.2	143	110	119
3	PSC	dysplasia	43	M	neg	Y	1.2	1.86	20	15.8	-	195	5.6	1.1	140	113	179
4	PSC	dysplasia	52	F	UC	N	0.3	0.89	2	13	4.4	319	5.3	0.9	143	30	31
5	PSC	dysplasia	42	M	UC	N	0.2	0.60	8	15.5	5.1	348	6.7	1.1	139	30	37
6	PSC	dysplasia	40	M	UC	Y	0.8	1.44	20	14.9	4.5	305	7.5	1.1	137	120	120
7	PSC	dysplasia	40	M	UC	-	1.6	1.75	16	13.9	4.9	244	6.2	1	138	197	339
8	PSC	cirrhosis	53	M	UC	Y	1	1.65	5	13.8	4.5	282	8	1	145	143	264
9	PSC	cirrhosis	32	M	UC	Y	4.5	6.41	7	12.2	3.9	60	2.2	1	140	136	128
10	PSC	cirrhosis	48	F	neg	Y	0.5	0.94	9	13.5	4.4	445	3.5	1.1	144	79	82
11	PSC	cirrhosis	33	F	UC	Y	5.1	6.22	23	10.4	3.3	68	3.6	1.4	134	121	89
12	PSC	cirrhosis	34	M	neg	N	1.4	4.51	6	10.6	4.5	65	2.5	1.1	141	44	26
13	PSC	cirrhosis	58	M	neg	N	2.6	8.31	22	12.2	3	81	4	1.5	132	107	85
14	PSC	cirrhosis	47	F	UC	N	5.2	5.88	25	12	3.5	148	7.5	1.6	136	271	214
15	PSC	cirrhosis	37	M	UC	Y	4.7	8.60	20	11.2	-	86	4.7	1.2	132	200	100
16	PSC	cirrhosis	32	M	UC	N	7.7	10.96	15	10.1	4.1	72	2.1	1.2	140	278	127
17	PSC	cirrhosis	66	M	neg	N	1.5	4.43	16	13.1	3.8	153	7.8	1.1	134	111	117
18	PSC	cirrhosis	67	M	UC	Y	1.9	7.20	20	11.6	-	184	6.4	1.2	136	179	82
19	PSC	cirrhosis	29	M	UC	Y	1.2	1.67	25	8.9	-	155	4.4	1.5	131	90	102
20	PSC	cholangitis	39	M	UC	N	0.2	0.43	10.00	15.3	4.9	466	14.7	1	135	40	61
21	PSC	cholangitis	38	M	UC	Y	0.8	1.38	5	14.3	-	220	5.4	0.9	141	87	119
22	PSC	cholangitis	55	F	UC	Y	1.1	1.81	5	12.9	3.9	246	7.9	1.1	143	93	132
23	PSC	cholangitis	54	F	neg	N	0.7	1.29	9	11.8	4.1	210	9.4	0.9	139	54	115
24	ALD	cirrhosis	64	F	neg	N	1.2	3.87	12	9.5	-	141	6.8	1.2	135	59	48
25	ALD	cirrhosis	55	M	neg	N	0.6	2.70	14	12.2	3.6	150	7.3	1.3	136	46	38
26	ALD	cirrhosis	70	M	neg	N	0.4	2.15	19	8.6	3.4	210	5.2	1.2	127	37	33
27	ALD	cirrhosis	70	M	neg	N	2	16.50	30	8.6	2.6	36	3.1	2.5	137	36	18
28	MASH	cirrhosis	62	F	neg	N	0.5	1.99	18	13.3	4.3	159	8.9	1.4	130	26	26
29	MASH	cirrhosis	58	M	neg	N	0.7	4.77	16	11.7	-	106	6.2	1.3	135	39	20
30	MASH	cirrhosis	56	M	neg	N	1.6	5.59	24	10.4	-	138	7.3	1.2	132	112	66

Patient ID	Disease	Transplant indication	Protein (g/L)	ALB (g/dL)	bilirubin (μmol/L)	CRP (mg/L)	γGT (U/L)	ALP (U/L)	Creatinine (μmol/L)	Dialysis (Y/N)	Dysplasia ERCP (Y/N)	Dysplasia TX	Cholangitis (Y/N)
1	PSC	dysplasia	72	45	7	0.6	100	134	67	N	Y	Y	N
2	PSC	dysplasia	70	46	14	1.1	537	255	82	N	Y	Y	Y
3	PSC	dysplasia	76	48	458	0.7	306	166	81	N	Y	Y	N
4	PSC	dysplasia	77	48	6	1.1	51	69	55	N	Y	Y	N
5	PSC	dysplasia	75	47	7	1.2	30	73	68	N	Y	Y	N
6	PSC	dysplasia	83	45	279	50	292	505	77	N	Y	Y	Y
7	PSC	dysplasia	78	42	181	17	327	565	71	N	Y	Y	Y
8	PSC	cirrhosis	74	44	37	4.6	1107	528	69	N	N	N	Y
9	PSC	cirrhosis	68	38	20	3.1	236	344	64	N	N	N	N
10	PSC	cirrhosis	69	43	55	1.7	229	325	66	N	N	N	Y
11	PSC	cirrhosis	52	27	248	23	30	281	53	N	N	N	N
12	PSC	cirrhosis	70	38	9	12	71	93	65	N	-	N	N
13	PSC	cirrhosis	74	22	122	17	137	188	56	N	N	N	N
14	PSC	cirrhosis	67	31	326	16	277	663	61	N	-	N	-
15	PSC	cirrhosis	70	32	90	46	450	665	71	N	N	N	N
16	PSC	cirrhosis	79	35	87	14	1123	722	50	N	N	N	N
17	PSC	cirrhosis	87	35	30	1	500	188	108	N	Y	N	Y
18	PSC	cirrhosis	79	36	100	8.9	250	494	116	N	N	N	Y
19	PSC	cirrhosis	95	27	189	65	153	346	33	N	N	N	Y
20	PSC	cholangitis	80	41	10	72	478	407	68	N	Y	N	Y
21	PSC	cholangitis	82	43	19	1.2	444	272	82	N	N	N	Y
22	PSC	cholangitis	82	44	16	10	832	393	51	N	N	N	N
23	PSC	cholangitis	75	41	14	5.7	1411	613	119	N	N	N	Y
24	ALD	cirrhosis	78	41	9	4	186	74	76	N	N	N	N
25	ALD	cirrhosis	54	26	22	28	73	112	95	N	N	N	N
26	ALD	cirrhosis	74	36	12	3.7	190	89	79	N	N	N	N
27	ALD	cirrhosis	55	44	98	12	56	89	167	N	N	N	N
28	MASH	cirrhosis	77	40	18	21	274	137	65	N	N	N	N
29	MASH	cirrhosis	59	31	29	13	172	137	103	N	N	N	N
30	MASH	cirrhosis	70	32	24	18	164	143	140	N	N	N	N

Supplementary Table 1 Abbreviations: ALB, albumin; ALD, alcohol-related liver disease; ALT, alanine aminotransferase; ALP, alkaline phosphatase; APRI, aspartate aminotransferase to platelet ratio index; AST, aspartate transaminase; CRP, C-reactive protein; ERCP, endoscopic retrograde cholangiopancreatography; FIB-4, fibrosis-4 index; γGT, gamma-glutamyl transferase; Hb, hemoglobin; IBD, inflammatory bowel disease; INR, international normalized ratio; leuko, leukocytes; MASH, metabolic dysfunction-associated steatohepatitis; MELD-Na, model for end-stage liver disease sodium, neg, negative; PSC, primary sclerosing cholangitis; TX, transplantation; UC, ulcerative colitis; UDCA, ursodeoxycholic acid

Supplementary Table 2. Differentially expressed genes between parenchyma subclusters pare.1-pare.5
https://riim-data.no/NoPSC_Liver_Atlas/supp.table.2-pare.deg.xlsx

Supplementary Table 3. Differentially expressed genes identified by Pseudobulk analysis between PSC and disease control parenchyma subclusters pare.1-pare.5
https://riim-data.no/NoPSC_Liver_Atlas/supp.table.3-pare.pseudo.deg.xlsx

Supplementary Table 4. Differentially expressed genes between fibrosis1 subclusters fibro1.1-fibro1-5
https://riim-data.no/NoPSC_Liver_Atlas/supp.table.4-fibro1.deg.xlsx

Supplementary Table 5. Differentially expressed genes identified by Pseudobulk analysis between PSC and disease control fibro1 subclusters fibro1.1-fibro1.5
https://riim-data.no/NoPSC_Liver_Atlas/supp.table.5-fibro1.pseudo.deg.xlsx

Supplementary Table 6. Differentially expressed genes between 9 major snRNA-seq cell clusters
https://riim-data.no/NoPSC_Liver_Atlas/supp.table.6-snRNAseq.deg.xlsx
